# Inhibition of GATA2 in prostate cancer by a clinically available small molecule

**DOI:** 10.1530/ERC-21-0085

**Published:** 2021-10-12

**Authors:** Salma Kaochar, Aleksandra Rusin, Christopher Foley, Kimal Rajapakshe, Matthew Robertson, Darlene Skapura, Cammy Mason, Karen Berman De Ruiz, Alexey Mikhailovich Tyryshkin, Jenny Deng, Jin Na Shin, Warren Fiskus, Jianrong Dong, Shixia Huang, Nora M Navone, Christel M Davis, Erik A Ehli, Cristian Coarfa, Nicholas Mitsiades

**Affiliations:** 1 Department of Medicine, Baylor College of Medicine, Houston, Texas, USA; 2 Dan L. Duncan Comprehensive Cancer Center, Houston, Texas, USA; 3 Department of Molecular and Cellular Biology, Baylor College of Medicine, Houston, Texas, USA; 4 Department of Education, Innovation, and Technology, Baylor College of Medicine, Houston, Texas, USA; 5 Division of Cancer Medicine, Department of Genitourinary Medical Oncology, The University of Texas Anderson Cancer Center, Houston, Texas, USA; 6 Avera Institute for Human Genetics, Sioux Falls, South Dakota, USA

**Keywords:** prostate cancer, castration-resistance, GATA2, Dilazep, c-MYC

## Abstract

Castration-resistant prostate cancer (CRPC) remains highly lethal and in need of novel, actionable therapeutic targets. The pioneer factor GATA2 is a significant prostate cancer (PC) driver and is linked to poor prognosis. GATA2 directly promotes androgen receptor (AR) gene expression (both full-length and splice-variant) and facilitates AR binding to chromatin, recruitment of coregulators, and target gene transcription. Unfortunately, there is no clinically applicable GATA2 inhibitor available at the moment. Using a bioinformatics algorithm, we screened *in silico* 2650 clinically relevant drugs for a potential GATA2 inhibitor. Validation studies used cytotoxicity and proliferation assays, global gene expression analysis, RT-qPCR, reporter assay, reverse phase protein array analysis (RPPA), and immunoblotting. We examined target engagement via cellular thermal shift assay (CETSA), ChIP-qPCR, and GATA2 DNA-binding assay. We identified the vasodilator* dilazep* as a potential GATA2 inhibitor and confirmed on-target activity via CETSA. Dilazep exerted anticancer activity across a broad panel of GATA2-dependent PC cell lines *in vitro* and in a PDX model *in vivo*. Dilazep inhibited GATA2 recruitment to chromatin and suppressed the cell-cycle program, transcriptional programs driven by GATA2, AR, and c-MYC, and the expression of several oncogenic drivers, including AR, c-MYC, FOXM1, CENPF, EZH2, UBE2C, and RRM2, as well as of several mediators of metastasis, DNA damage repair, and stemness. In conclusion, we provide, via an extensive compendium of methodologies, proof-of-principle that a small molecule can inhibit GATA2 function and suppress its downstream AR, c-MYC, and other PC-driving effectors. We propose GATA2 as a therapeutic target in CRPC.

## Introduction

In 2021, prostate cancer is predicted to cause the death of 34,130 US men, making it the second leading cause of cancer death in American men, after lung cancer (Siegel *et al.* 2021). Importantly, this represents a 30.6% increase in mortality compared to 2016, highlighting the unmet need for effective therapies. Progression of metastatic PC after first-line endocrine therapy is inevitable and the resulting castration-resistant PC (CRPC) is incurable and highly lethal. Second-line therapies provide small extensions of survival, while immunotherapy has not delivered in this area the significant benefits it afforded to patients with other malignancies. Consequently, new therapeutic targets and treatment approaches are needed for CRPC.

The transcription factor (TF) GATA2 has been identified as an important driver of PC, including CRPC. GATA2, one of six members of the GATA family of TFs, has been primarily implicated in driving developmental and differentiation processes, particularly in hematopoietic stem cells (Tsai *et al.* 1994, Tsai & Orkin 1997, Bresnick *et al.* 2010). GATA2 is the most highly expressed GATA family member in normal and malignant human prostate, while in the mouse, both GATA2 and GATA3 are highly expressed in the anterior and dorsal-lateral prostate lobes (Xiao *et al.* 2016). We have generated prostate-specific double *Gata2* and *Gata3* knockout (KO) mice and found that they exhibit decreased prostate-to-body weight ratio and dramatically reduced expression of the mRNAs for the AR-target genes *Probasin* and *Nkx3.1* (Xiao *et al.* 2016). In human PC cells, silencing *GATA2* results in inhibition of cell proliferation, AR expression, and AR signaling. The effects of GATA2 can be exerted at several steps of the AR signaling axis, including functioning as a pioneer factor (that facilitates AR to access chromatin and initiate transcription (Wang *et al.* 2007), inducing AR gene expression (He *et al.* 2014, Wu *et al.* 2014) and post-translationally promoting the activity of both full-length and splice-variant AR by enhancing recruitment of steroid receptor coactivators to AR (He *et al.* 2014, Zhao *et al.* 2016). AR-independent growth-promoting effects of GATA2 have also been reported (Vidal *et al.* 2015). As high GATA2 expression and transcriptional activity are linked to poor prognosis in PC (Chiang *et al.* 2014, He *et al.* 2014), and GATA2 being considered a transcriptional partner and coactivator for AR, GATA2 is poised as a promising therapeutic target for the inhibition of advanced CRPC.

We previously demonstrated that the small molecule K-7174, which has been reported to be a potential GATA inhibitor within the context of endothelial cell adhesion (Umetani *et al.* 2000), exerts anticancer activity against GATA2-dependent PC cells (He *et al.* 2014). However, K-7174 is not being developed further by its manufacturer/patent holder, and, consequently, it has no path to the clinic. This led us to examine for agents with a structural similarity that could be used clinically. Via *in silico* screen, we identified dilazep, a drug that has been used as a vasodilator (mechanistically works as an adenosine reuptake inhibitor (Deguchi *et al.* 1997)) in patients with hypertension, cardiovascular, and renovascular disorders in Japan and several European countries (Sambhi *et al.* 1989). Our studies in PC cells demonstrate that dilazep is able to inhibit proliferation and AR signaling in both androgen-dependent cells and castration-resistant PC cell lines *in vitro* and is active in an *in vivo* patient-derived xenograft (PDX) model. We also validated, via global gene expression profiling, RT-qPCR and reporter assay, its capacity to inhibit the GATA2 transcriptional program. Dilazep suppressed the expression of AR, c-MYC, FOXM1, CENPF, EZH2, and several other PC drivers. Using cellular thermal shift assay (CETSA), ChIP-qPCR, and GATA2 DNA-binding assay, we further confirmed GATA2 target engagement by dilazep and inhibition of GATA2 recruitment to chromatin and DNA. Our findings provide proof of principle that GATA2 can be effectively targeted by small molecules in PC for therapeutic purposes.

## Materials and methods

### Structure-based prediction

In order to identify compounds with similar properties as K-7174, we utilized SuperPred (https://prediction.charite.de/), an algorithm that was designed to look for similar therapeutic compounds based on a 2D, fragment, and 3D similarity search pipeline (Nickel *et al.* 2014). The input compound is screened against 2650 drugs that have anatomical therapeutic chemical (ATC) classification codes assigned. Therefore, only those drugs can be displayed as similar drugs. The resulting score is based on the output of the similarity pipeline, which compares structural fingerprints as well as 3D structures using a superposition algorithm.

### Cell culture

LNCaP cells (American Type Culture Collection (ATCC)) were cultured in RPMI-1640 (Gibco (ThermoFisher)) supplemented with 10% fetal bovine serum (FBS) (Gibco). LNCaP-Abl cells (henceforth referred to as Abl cells) (Culig *et al.* 1999) (a kind gift from Dr Zoran Culig, Innsbruck Medical University, Inssbruck, Austria) were cultured in phenol-red free RPMI-1640 media (Gibco) supplemented with 10% charcoal-stripped serum (CSS). LNCaP MDV-3100/enzalutamide-resistant (MDVR) cells were generated by continually culturing parental LNCaP cells as previously described, with the addition of 25 μM MDV-3100 (enzalutamide), for more than a year (enzalutamide concentration was decreased to maintenance of 10 μM during experiments). 22Rv1 cells (ATCC) were cultured in RPMI-1640 with 10% FBS. LAPC4 cells (ATCC) were cultured in Iscove’s Modified Dulbecco’s Media (IMDM, Life Technologies) plus 15% FBS, 1 nM R1881, and 2 mM of L-glutamine. PC-3 cells (ATCC) were cultured in DMEM/F12 (F-12 Nutrient Medium, Life Technologies) with 10% FBS. RWPE-1 epithelial cells (ATCC) derived from the peripheral zone of a histologically normal adult human prostate and immortalized via human papilloma virus 18 were cultured in keratinocyte serum-free medium according to the instructions of ATCC. All cells culture media also contained penicillin/streptomycin (P/S; 100 units/mL; Gibco). Cells were maintained in a 37°C incubator with 5% CO_2_. All cell lines were authenticated by STR fingerprinting on an annual basis and used within six passages after thawing. Cell lines were tested for mycoplasma contamination prior to experimental use.

### MTT assay

Cells were seeded in 24-well plates and following 24 h of attachment treated with dilazep (Santa Cruz Biotechnologies) (or vehicle control) at the indicated concentrations for 96 h, at which point MTT (Sigma-Aldrich) was added to a final concentration of 50 μg/mL and incubated at 37°C for 2 h. The precipitated crystals were dissolved with 92% isopropanol/8% 1 N HCl. The optical density was calculated as the difference between the absorbance at 570 nM and the absorbance at 630 nM and normalized to the respective controls.

### 5-Ethynyl-2’-deoxyuridine (EdU) incorporation/cell proliferation assay

22Rv1 (1 × 10^5^ cells/well) and LNCaP cells (2.5 × 10^5^ cells/well) were seeded in 12-well plates. The following day, the cells were treated with 0, 20, or 50 µM dilazep for 24 h. Experiments were performed in triplicates. Before harvesting, the cells were incubated in the presence of 10 µM EdU as a thymidine nucleoside analog for 90 min, to allow incorporation of the compound during the S-phase of a cell cycle. Newly synthesized EdU-labeled DNA was fluorescently labeled through a click chemistry reaction with the Alexa Fluor® 488 (AF488) conjugated azide. AF488-labeled azide was reacted with the incorporated EdU according to the manufacturer protocol (Click-&-Go EdU 488 Flow Cytometry Assay Kit; Click Chemistry Tools). Cells were labeled with DAPI to evaluate DNA content. BD FacsCanto flow cytometer was used for determining the percentage of S-phase cells in the population. The data were analyzed in FlowJo.

### Gene expression profiling after treatment with dilazep

Cells were treated with 50 µM dilazep for 48 h. Total RNA was extracted using Trizol (Life Technologies) and purified with the RNeasy Mini Kit (Qiagen) following the manufacturer’s instructions. RNA was reverse transcribed and the microarray hybridization was performed using the Illumina Gene Expression Sentrix BeadChip HumanHT-12_V4 (Illumina) at the Laboratory for Translational Genomics at Baylor College of Medicine, as previously described (Geng *et al.* 2014). Each experimental condition (cell line/treatment) was studied in triplicate.

### Gene expression profiling after treatment with siRNA

Cells were transfected with Stealth siRNA (25 nM, LifeTechnologies, ThermoFisher Scientific) against GATA2 (clone: HSS104003), or non-target control (siNT), using Lipofectamine RNAiMax (LifeTechnologies) according to the manufacturer’s instructions and as previously described (He *et al.* 2014). RNA was extracted 72 h post-transfection using Trizol (LifeTechnologies) according to the manufacturer’s instructions. RNA was submitted for library preparation and quantification at the Avera Institute for Human Genetics (Sioux Falls, SD), where it was processed as previously (Kaochar *et al.* 2018). Each experimental condition (cell line/treatment) was studied in triplicate.

### Gene set enrichment analysis

All microarray data were normalized by using the Bioconductor lumi (Du *et al.* 2008) package using the R statistical system. Gene-expression differences were inferred using *t*-test and imposing a fold change > 1.25 or < 1/1.25 (*P* < 0.05). Gene set enrichment analysis (GSEA) was carried out using the GSEA software package (Subramanian *et al.* 2005) to assess the degree of similarity among the studied gene signatures, as previously described (Geng *et al.* 2014, 2017). In addition, we utilized a signature derived from genes differentially expressed between metastatic CRPC and primary, hormone-naïve PC patient specimens (Cai *et al.* 2013). We also utilized a recently described MYC/RAS co-activation signature (META-16) associated with prostate cancer metastasis (Arriaga *et al.* 2020). Moreover, we performed ingenuity pathway analysis (Qiagen). For analysis of c-MYC transcriptomic signatures in prostate models (described in detail in Geng *et al.* 2017), we used three publicly available gene expression datasets: (1) following knockdown of c-MYC via siRNA in LNCaP, DU145, and PC3 PC cells (Koh *et al.* 2011), (2) upon overexpression of c-MYC for 5 or 12 h in LNCaP cells (GSE51384 and Barfeld *et al.* 2015), and (3) upon c-MYC overexpression in epithelial cells isolated from the mouse ventral prostate (GSE37428 and Ju *et al.* 2013).

### Comparison of the transcriptional program of dilazep with GATA2 activity score, AR activity score, and c-MYC activity score in PC patient cohorts

We applied the gene signature derived from treatment of LNCaP cells with dilazep, as well as the previously published footprints of GATA2 (LNCaP+GATA2 siRNA, GSE63539), AR (LNCaP+AR siRNA, GSE63539) and c-MYC (overexpression of c-MYC for 12 h in LNCaP cells (GSE51384 and Barfeld *et al.* 2015), and knockdown of c-MYC via siRNA in LNCaP (Koh *et al.* 2011)) to three previously reported cohorts of human primary PC specimens collected via prostatectomy: Taylor *et al.* (2010), Cai *et al.* (2013), and the cancer genome atlas (TCGA) (https://tcga-data.nci.nih.gov/tcga/). Within each dataset, we utilized the expression of each gene to calculate its respective z-score for each sample, relative to the normal prostate gland specimens available in that cohort. We computed the sum z-score for each sample (the z-scores of downregulated genes were subtracted from the z-scores of upregulated genes), as described previously (Taylor *et al.* 2010). Finally, for each pair of signatures, we plotted the cumulative z-scores on the x and y axis and computed the Pearson correlation coefficient R and associated *P*-value using the R statistical system.

### Reverse phase protein array analysis

RPPA analysis was performed as previously described (Chang *et al.* 2015, Creighton & Huang 2015). Briefly, cells were grown in six-well dishes and treated with 50 μM dilazep for 48 h. Cells were washed, lysed, quantified, and submitted to the Baylor College of Medicine Antibody-based Proteomics Core for analysis. Each lysate was run in technical triplicates, with three biological replicates run per group. The fluorescence-labeled slides were scanned with a Molecular Devices GenePix 4400 AL and the intensity data were extracted with GenePix Pro 7.0. Intensity for each spot was obtained after subtracting the local background and group-based normalization (Chang *et al.* 2015) was used to adjust for total protein variation, background, and non-specific labeling. Subsequent statistical analysis were performed on the median of the technical triplicate in each sample. Proteins with maximum intensity less than 200 were removed from the subsequent analysis and significantly altered proteins between different experimental conditions were determined using the Student’s *t*-test with *P*-value < 0.05 and fold change > 1.25 or < 1/1.25.

A complete list of antibodies used for RPPA is presented in Supplementary Table 1 (see section on supplementary materials given at the end of this article).

### Immunoblotting

Cells were lysed in RIPA lysis buffer (25 mM Tris-HCl, 150 mM NaCl, 1% IGEPAL-630, 0.5% sodium deoxycholate, 0.1% SDS, 1 mM EDTA, 5% glycerol, protease inhibitor tablets (Roche), and phosphatase inhibitor tablets (Roche)). Lysates were sonicated for 1 min (BioRuptor, Diagenode (Belgium)). Total protein was quantified via Biorad DC Kit (Biorad). Total protein lysate (40 μg) was loaded into 8.5% polyacrylamide gels or NuPage 4–12% Bis-Tris gels and transferred as previously described (He *et al.* 2014). Images were acquired using an infrared scanner (Odyssey (LiCoR), Lincoln, NE) and analyzed with the corresponding ImageStudio software (LiCoR). Antibodies used were: AR (Cell Signaling, #3202), GATA2 (Cell Signaling, #4595), GATA2 (Abclonal, #A0677), Vinculin (Santa Cruz Biotechnologies, sc-25336), c-MYC (Cell Signaling, #9402), and β-actin (Santa Cruz, sc-8432 or Invitrogen #MA1-140).

### Quantitative real-time reverse transcription PCR (RT-qPCR)

RNAs were purified using Rneasy Mini Kit (Qiagen) and cDNA synthesis was performed using cDNA RT Kit (Applied Biosystems). mRNA expression was measured using a real-time detection system (Applied Biosystems StepOnePlus™) in 96-well optical plates using PerfeCTa™qPCR FastMix™ (Quanta Biosciences). 18S was used as an endogenous control. The primer sequences were: CCNA2, 5’-TTGTAGGCACGGCTGCTATGCT-3’ and 5’-GGTGCTCCATTCTCAGAACCTG-3’; CENPF, 5’-AGCACGACTCCAGCTACAAGGT-3’ and 5’-CATCATGCTTTGGTGTTCTTTCTG-3’; AURKA, 5’-GCAACCAGTGTACCTCATCCTG-3’ and 5’-AAGTCTTCCAAAGCCCACTGCC-3’; MYC, 5’-CCTGGTGCTCCATGAGGAGAC-3’ and 5’-CAGACTCTGACCTTTTGCCAGG-3’; RAD51AP1, 5’-CTTCTGGAAGGCAGTGATGGTG-3’ and 5’-AGAGAAGTCTTCGTCATTATCCTC-3’. All analyses were performed in triplicate, and statistical evaluation was performed via *t*-test.

### Cellular thermal shift assay (CETSA)

To analyze the interaction between GATA2 and dilazep, CETSA was performed as described previously (Jafari *et al.* 2014), with minor modifications. Briefly, 22Rv1 cells were incubated with vehicle (DMSO) or dilazep (50 μM) for 1 h. After treatment, cell pellets were washed and resuspended in PBS buffer containing protease and phosphatase inhibitors. The cell suspension was aliquoted into PCR tubes and heated for 3 min in the Veriti 96-well thermal cycler to 61, 64, and 67°C. Subsequently, cells were lysed by two repeated freeze-thaw cycles with liquid nitrogen. The cell lysates were briefly vortexed in the tubes and then centrifuged at 20,000 ***g*** for 20 mins at 4°C. The supernatant was analyzed by SDS-PAGE (25 μL of lysate from each tube), followed by immunoblotting analysis. The protein levels of GATA2 were normalized to the corresponding levels of vinculin control for each condition.

### GATA2 DNA binding assay

The DNA binding assay for GATA2 was performed using the GATA2 transcription factor activity assay kit (RayBiotech, TFEH-GATA2) and nuclear extracts from LNCaP cells (cultured in RPMI supplemented with 10% FBS and 5 nM of R1881) treated with DMSO (control) or dilazep (50 µM or 100 µM) for 16 h, according to manufacturer’s protocol. Briefly, this assay uses a double stranded DNA-coated plate with canonical GATA2 binding sequences (GATA motif ATCWGATA (W = A/T)) to detect active GATA2 in nuclear extracts (50 µg per well) following short incubation. Subsequently, primary antibody against GATA2 is used to recognize the GATA2-DNA complex and a HRP-conjugated secondary antibody is used for detection. After washing away any unbound antibody, signal absorbance is captured using a spectrophotometric plate reader at 450 nm. Nuclear extract was prepared using the Nuclear and Cytoplasmic Extraction Kit (NE-PER™ Nuclear and Cytoplasmic Extraction Reagents, Catalog number: 78833, ThermoFisher Scientific) according manufacturer’s instructions.

### GATA2 ChIP-PCR

LNCaP cells (1.5 × 10^7^) were plated in 15 cm dishes and kept for 24 h in RPMI1640 supplemented with 10% FBS, to allow the cells to attach to the dish. Cells were treated with 5 nM R1881 and either dilazep (10 μM) or DMSO for 16 h. Cross-linking of proteins to DNA was performed by adding formaldehyde drop-wise directly to the media to a final concentration of 1% and rotating gently at room temperature (RT) for 15 mins. Glycine was added to a final concentration of 125 mM to the media, and then cells were incubated for 5 mins at RT, followed by rinsing twice with cold PBS. Cells were scraped off in cold PBS with protease inhibitor cocktail (cOmplete™, Roche), cell suspension was transferred into 15 mL tubes and centrifuged for 5 mins at 4°C, 750 ***g***. The pellet was resuspended in ChIP lysis buffer with protease inhibitors (1000 μL PBS per 1 × 10^7^ cells) and incubated for 10 mins on ice. Lysates were sonicated with Bioruptor Pico sonication device (Diagenode) to shear DNA to an average fragment size of 200–500 bp. Samples were diluted 1:5 with ChIP dilution buffer and incubated either with 10 μg of GATA2 antibody (sc-9008X SantaCruz) or IgG and rotated at 4°C overnight. Next, protein A/G agarose beads (sc-2003, SantaCruz) was added to all samples and rotated for 6 h at 4°C. Samples were washed several times with various salt wash buffers. DNA was eluted using elution buffer, reverse crosslinked, and treated with RNase A and proteinase K for 1 h. Subsequently, DNA was purified using Purelink PCR cleaning kit and analyzed by real-time quantitative PCR using Power SYBR® Green PCR master mix (Applied Biosystems). Sequences of ChIP primers used: GATA2 c-MYC enh (amplified fragment chr8:127852571+127852674), forward primer: TCGTAGGGTTGGTGTTTGAATG, reverse primer: CAAGGGCTTAACCTGCCTTAAT; KLK3 (amplified fragment chr19:50850923+50851007), forward primer: GCCTGGATCTGAGAGAGATATCATC, reverse primer: ACACCTTTTTTTTTCTGGATTGTTG; gene desert (amplified fragment chr17:79882948+79883067), forward primer: TTGACTGCAGGGAGTCAGTG, reverse primer: GATTTGTGGGGGTGATGAAG.

### Reporter assay

22Rv1 cells were seeded in 96-well plates (10,000 per well) in RPMI1640 with 10% FBS and incubated at 37°C, 5% CO_2_, to allow attachment. Cells were transfected with a reporter vector (pGL3/PSA61-Luc (Cleutjens *et al.* 1997)) and vectors encoding for Renilla luciferase (pGL4.75, Promega) and GATA2 (pcDNA3-GATA2) using jet PRIME transfection kit (Polyplus) according to the manufacturer protocol. After 24 h, cells were treated with 5 nM R1881 and either 10 µM dilazep or DMSO for 24 h. Luciferase activity in cells was measured with Dual-Luciferase Reporter Assay System (Promega) according to the manufacturer’s protocol. In brief, after treatment, the cells were washed with PBS and lysed with 20 µL of passive lysis buffer. Lysates were transferred into a 96-well white opaque plate, and firefly luciferase and Renilla luciferase activity was measured using SynergyH1 plate reader (Biotek). Firefly reagent was injected to each well, and after quantifying the firefly luminescence, this reaction was quenched, and the Renilla luciferase reaction was simultaneously initiated by adding Stop & Glo® Reagent to the same well. Subsequently, Renilla luciferase activity was measured. Firefly luciferase activity was normalized to Renilla luciferase activity in each well. Assays were performed in triplicates.

#### In vivo studies

All animal experiments and procedures were performed in compliance with ethical regulations and the approval of the Baylor College of Medicine Institutional Animal Care and Use Committee (IACUC). Six-week-old CB17SCID (CB17/Icr-*Prkdc^scid^*/IcrIcoCrl) immunodeficient male mice were purchased from Charles Rivers Laboratories (Strain code 236) and used as transplant recipients. All mice were housed in a pathogen-free animal barrier facility under constant humidity and temperature, with 12 h light:12 h darkness cycles and monitored daily. We used a PC patient-derived xenograft (PDX) model, MDA-PCa-337A, generated by Dr Nora Navone at MD Anderson Cancer Center from a liver metastasis. To establish tumors, a 3 × 3 × 3 mm fragment of tumor was coated in Matrigel and implanted subcutaneously into the left flank of castrated SCID mice. Treatment was initiated one day after implantation. Seven animals were used per group. Dilazep was dissolved in molecular grade water at 200 mg/mL and administered intraperitoneally at 50 mg/kg daily 5 days per week (M–F). Tumor growth was monitored weekly by caliper measurements. Mice were weighed and observed regularly throughout the study for signs of illness or distress related to tumor growth and/or drug toxicity. Statistical analysis was performed by two-way ANOVA. All mice were euthanized by CO_2_ asphyxiation followed by cervical dislocation once tumors in untreated mice reached near protocol-defined tumor size limits (15 mm max diameter).

### Statistical analysis

Unless otherwise indicated, statistical analysis between groups was performed via the Student’s *t*-test. For the *in vivo* studies, we used two-way ANOVA.

## Results

### *In silico* prediction of a clinically available drug with properties similar to K-7174

Previously, we reported K-7174 as a potent small molecule inhibitor of GATA2 driven transcriptional program in PC cells (He *et al.* 2014). Unfortunately, K-7174 has no clear path to the clinic. Thus, we screened for alternative, structurally related compounds *in silico*, using the SuperPred algorithm (http://prediction.charite.de/) (Nickel *et al.* 2014) and identified dilazep (C_31_H_44_N_2_O_11_, MW 604.7 g/mol), a vasodilator, as a clinically available drug with potential inhibitory activity against GATA2 (Supplementary Fig. 1).

### Dilazep inhibits GATA2-dependent PC cell proliferation

**Figure 1:**
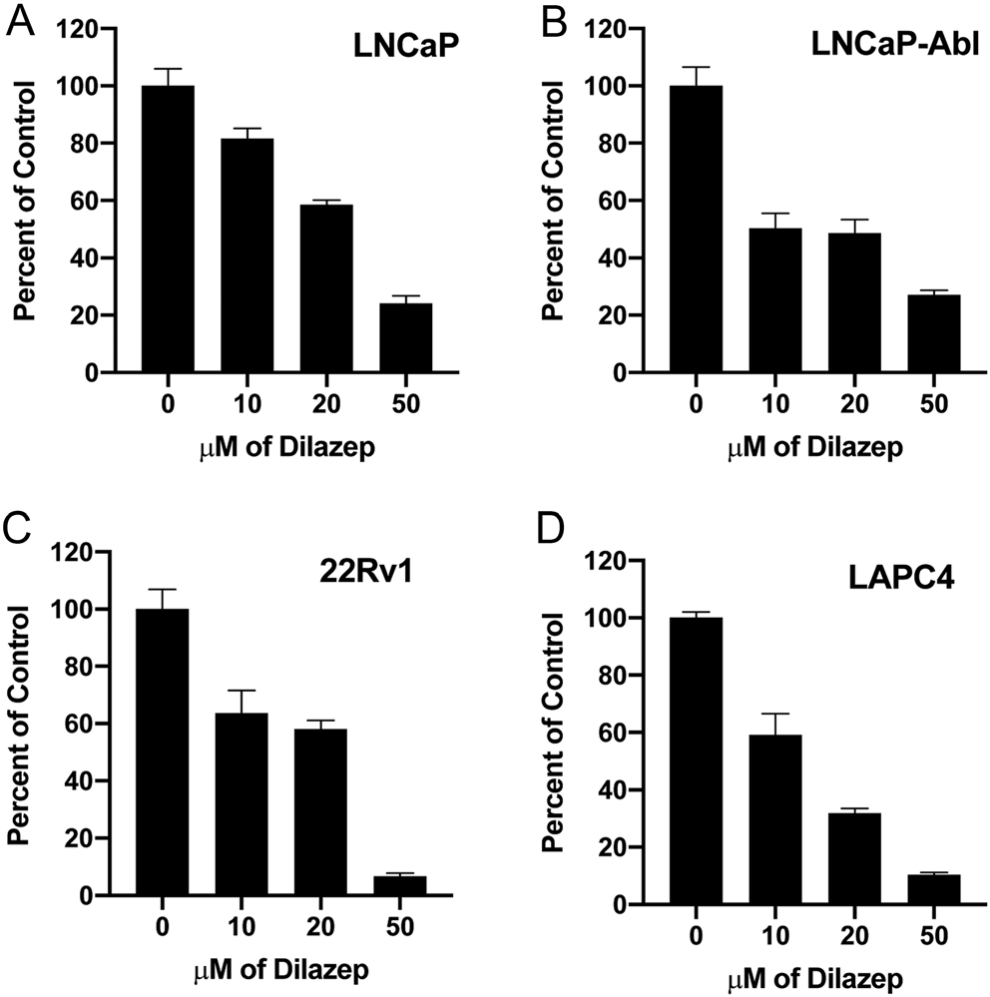
Dilazep inhibits proliferation of PC cells: MTT assay in PC cell lines after treatment with increasing concentrations of dilazep (0–50 µM) for 96 h. OD was calculated as absorbance at 570 nM – absorbance at 630 nM and normalized to the respective vehicle controls. Data are shown as average ± s.d. The IC50 for dilazep was generally in the range of ~10–20 µM.

We first examined the anticancer activity of dilazep against a panel of GATA2-dependent CRPC cell lines using MTT viability assays (Fig. 1): AR-positive, androgen-sensitive LNCaP, and LAPC4 (previously demonstrated to be GATA2-dependent (He *et al.* 2014)); AR-positive, androgen insensitive (LNCaP)-Abl and (AR-variant harboring) 22Rv1 (previously demonstrated to be GATA2-dependent (He *et al.* 2014)), and enzalutamide-resistant (MDVR) cells. The MDVR cells express higher levels of several cancer drivers compared to parental LNCaP cells (Supplementary Fig. 2), including SOX9 (an important transcription factor and CRPC driver (Wang *et al.* 2008, Ma *et al.* 2016)), YAP1 (Kuser-Abali *et al.* 2015), HER3 (Soler *et al.* 2009), phospho-Akt, SRC-3 (an important transcriptional coactivator (Zhou *et al.* 2005, Geng *et al.* 2013, Foley & Mitsiades 2016)), and EZH2 (Varambally *et al.* 2002, Bryant *et al.* 2007, Yu *et al.* 2010, Xu *et al.* 2012). MDVR cells also exhibited increased levels of LC3B/ATG3 and decreased levels of LC3A, suggesting a role for autophagy, in agreement with prior reports that increased autophagy may serve as a survival mechanism of resistance to enzalutamide (Nguyen *et al.* 2014, Kranzbuhler *et al.* 2019). MDVR cell proliferation was GATA2-dependent (Supplementary Fig. 3A) and sensitive to dilazep (Supplementary Fig. 3B). Inhibition of proliferation by dilazep was significantly lower in PC-3 and RWPE-1 cells (Supplementary Fig. 3C), both of which are AR-negative and, as we have previously demonstrated (He *et al.* 2014), GATA2-independent.

The results of the MTT assay were confirmed with the EdU incorporation assay (Supplementary Fig. 3D, E, F and G), which showed concentration-dependent inhibition of cell proliferation, evidenced by reduced percent of cells in S-phase in 22Rv1 and LNCaP cells.

### Dilazep targets cell cycle-related signaling in both androgen-dependent and CRPC cell lines

**Figure 2:**
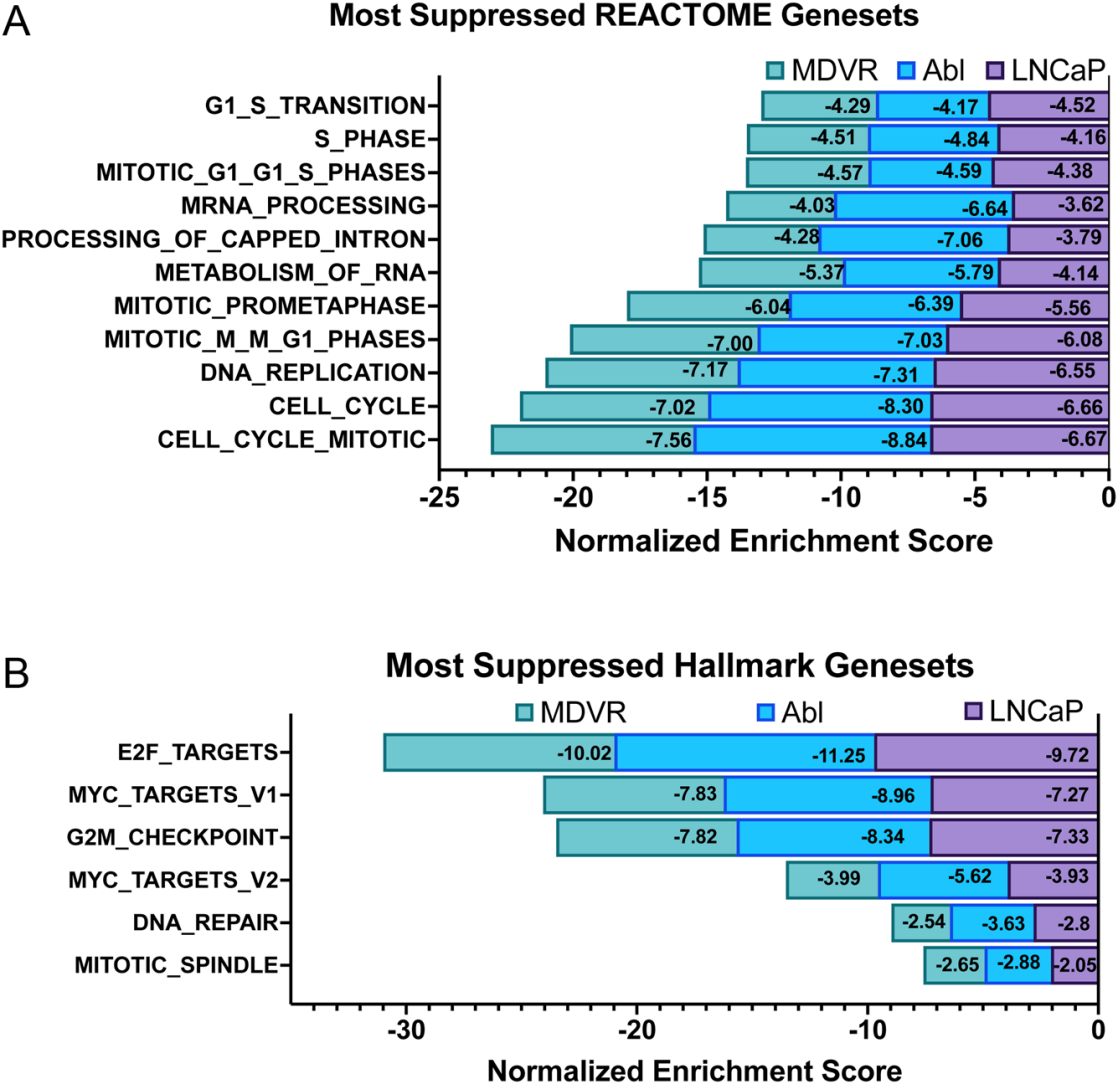
Dilazep suppresses cell cycle and the c-MYC transcriptional program. (A) Using GSEA, we analyzed our dilazep signatures (derived from treatment with 50 µM dilazep for 48 h) and found that, across all three PC cell lines tested, the most suppressed REACTOME gene sets were related to cell-cycle, mitosis and DNA replication. (B) Using GSEA, we analyzed our dilazep signatures and found that, across all three PC cell lines tested, the most suppressed HALLMARK gene sets were related to cell-cycle progression (E2F Targets and G2/M Checkpoint), downstream targets of c-MYC signaling (Myc Targets v1 and Myc Targets v2), and DNA Repair, highlighting dilazep as a potentially beneficial compound for inhibition of PC growth, regardless of androgen-dependence. All *P* < 0.001. A full color version of this figure is available at https://doi.org/10.1530/ERC-21-0085.

In order to characterize the molecular pathways affected by dilazep in PC cells, we treated the androgen-dependent LNCaP cells, the androgen-independent Abl, and the MDV-3100-resistant MDVR cells with dilazep, followed by global gene expression analysis. GSEA of these transcriptional profiles against the Molecular Signature Database (MSigDB) revealed that the most suppressed (top enriched terms) were associated with cell cycle, mitosis, DNA replication, E2F signaling, c-MYC signaling, and DNA repair (Fig. 2A, B and Supplementary Fig. 4A). We also found suppression of gene sets related to stemness, such as the transcriptional programs of Nanog, Sox2, Oct4, and other stem cell programs (Supplementary Fig. 4B). The transcriptional signature caused by dilazep treatment (50 µM for 48 h) was highly concordant between our three cell line models. A list of selected genes involved in cell-cycle regulation, cell survival, metabolism, DNA replication, recombination, and repair, and their fold-change (FC) in expression after dilazep treatment, are shown in Supplementary Fig. 5A. Importantly, the mRNAs for c-Myc itself, FOXM1, CENPF, EZH2, UBE2C, RRM2, and several other PC drivers were significantly suppressed by dilazep.

Confirmatory results obtained by RT-qPCR are shown in Supplementary Fig. 5B.

### Gene expression analysis reveals that dilazep suppresses GATA2- and AR-driven signaling in PC cells

**Figure 3:**
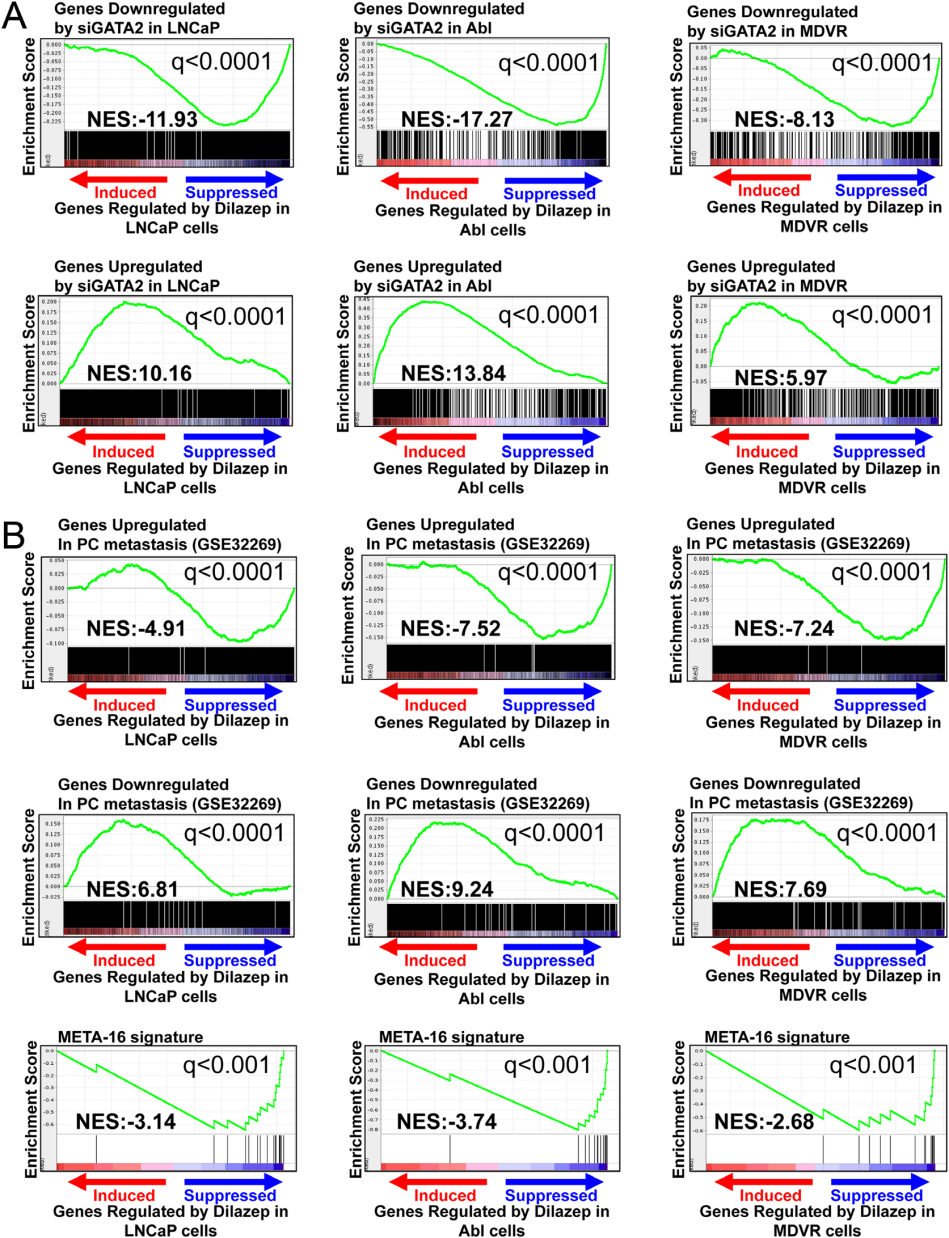
(A) Dilazep suppresses GATA2-driven signaling in PC cells. We compared, via GSEA, the transcriptional footprint of dilazep (50 µM for 48 h) with that of GATA2 siRNA in LNCaP, Abl, and MDVR cells. In each cell line, genes downregulated by GATA2 siRNA were strongly enriched among the genes suppressed by dilazep, while genes upregulated by GATA2 siRNA were strongly enriched among the genes induced by dilazep in the same cell line. This provides evidence that dilazep indeed suppresses that GATA2 transcriptional program, in both androgen-dependent and castration-resistant PC cells. (B) Dilazep suppresses the metastatic program of PC cells. We compared, via GSEA, our dilazep-derived signatures in LNCaP, Abl, and MDVR cells to a signature derived from genes differentially expressed between metastatic CRPC and primary, hormone-naïve PC patient specimens. We found that genes preferentially expressed in metastatic CRPC tissues were strongly suppressed by dilazep in all three cell lines tested, while genes downregulated in metastatic CRPC tissues were strongly enriched among the genes induced by dilazep, supporting that dilazep suppresses the metastatic program of PC cells. Moreover, we found that dilazep suppressed, in all three lines tested, a recently described MYC/RAS co-activation signature (META-16) (Arriaga *et al.* 2020) that is associated with prostate cancer metastasis. A full color version of this figure is available at https://doi.org/10.1530/ERC-21-0085.

Next, we compared the transcriptional footprint of dilazep with that of GATA2 siRNA in LNCaP (GSE63539, previously reported by our group (He *et al.* 2014)), Abl, and MDVR cells (generated as part of this current study). We found that the transcriptional footprint of dilazep was strongly concordant with that of GATA2 siRNA both in androgen-dependent and castration-resistant PC cells. In each cell line, genes downregulated by GATA2 siRNA were strongly enriched among the genes suppressed by dilazep, while genes upregulated by GATA2 siRNA were strongly enriched among the genes induced by dilazep in the same cell line. This validates our bioinformatics prediction and provides evidence that dilazep indeed suppresses the GATA2 transcriptional program, in both androgen-dependent and castration-resistant PC cells (Fig. 3A).

Having previously demonstrated that GATA2 is necessary for AR expression and transcriptional activity in PC cells (He *et al.* 2014), we examined whether dilazep can inhibit AR signaling in PC cells. For that, we compared the transcriptional footprint of dilazep with that of AR siRNA in LNCaP (GSE63539, previously reported by our group (He *et al.* 2014)) and Abl (GSE11428, by Wang *et al.* 2009). We found that the transcriptional footprint of dilazep was strongly concordant with that of AR siRNA both in androgen-dependent and castration-resistant PC cells. In each cell line, genes downregulated by AR siRNA were strongly enriched among the genes suppressed by dilazep, while genes upregulated by AR siRNA were strongly enriched among the genes induced by dilazep in the same cell line. This provides evidence that dilazep indeed suppresses the AR axis, not only under androgen-dependent conditions but also in CRPC cells (Supplementary Fig. 6).

### Dilazep suppresses the metastatic program of PC cells

We also compared our dilazep-derived signatures to a signature derived from genes differentially expressed between metastatic CRPC and primary, hormone-naïve PC patient specimens (Cai *et al.* 2013). Importantly, we found that genes preferentially expressed in metastatic CRPC tissues were strongly suppressed by dilazep in all three cell lines tested, while genes downregulated in metastatic CRPC tissues were strongly enriched among the genes induced by dilazep, supporting that dilazep suppresses the metastatic program of PC cells (Fig. 3B). Moreover, we found that dilazep suppressed, in all three lines, a recently described MYC/RAS co-activation signature (META-16) (Arriaga *et al.* 2020) that is associated with prostate cancer metastasis (Fig. 3B).

### Dilazep suppresses the c-MYC transcriptional program in PC cells

**Figure 4:**
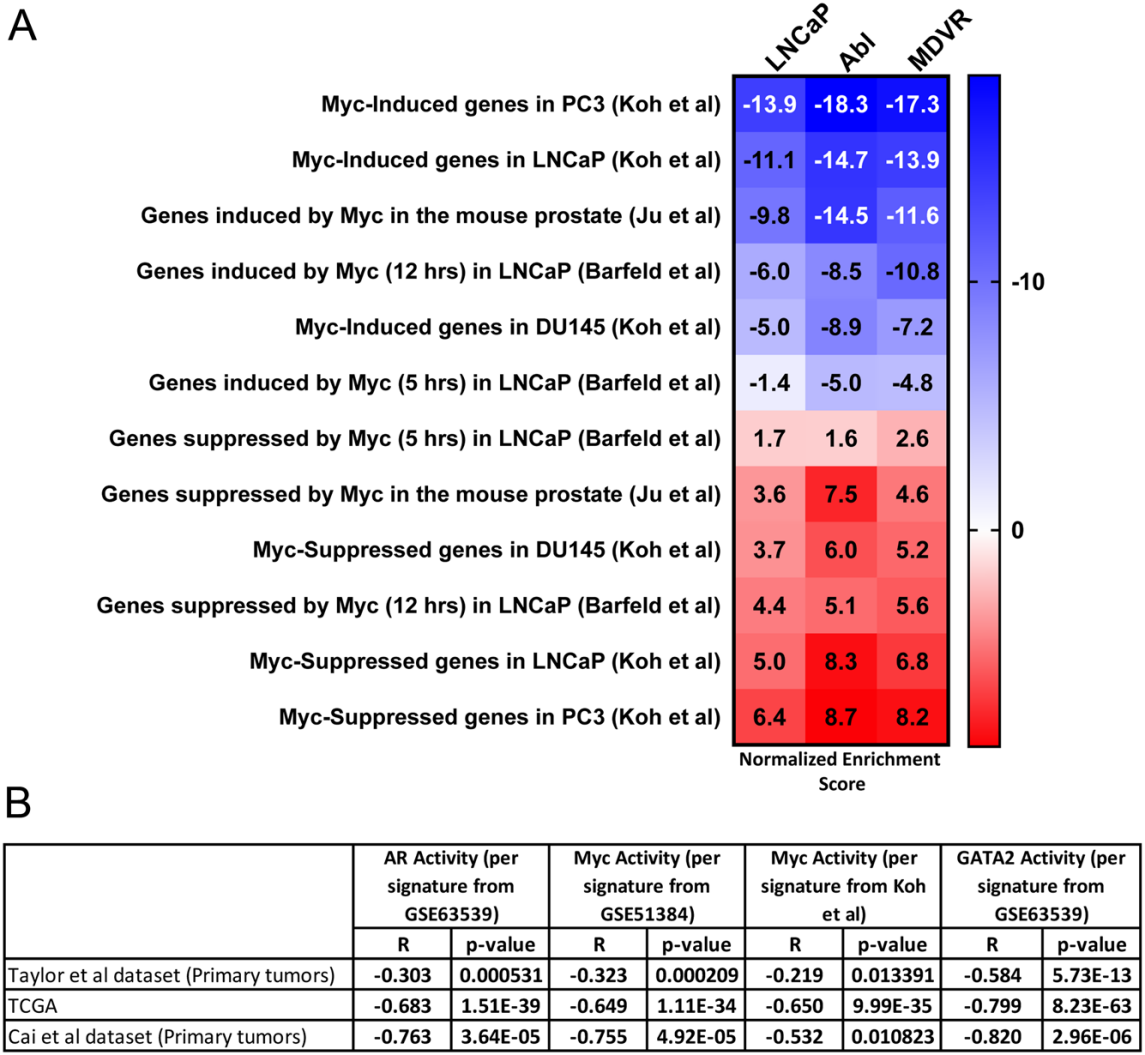
(A) Dilazep suppresses the c-MYC transcriptional program. Using GSEA, we compared our signatures of dilazep treatment in our three PC cell lines against publicly available prostate-specific signatures of c-MYC activity. We found that the gene programs induced by c-MYC are strongly suppressed by dilazep in all three cell lines for, while genes suppressed by c-MYC are strongly induced by dilazep. These results demonstrate that dilazep potently suppresses c-MYC activity in PC cells. All *P* < 0.001. (B) The dilazep transcriptional program correlates with decreased GATA2 activity score, AR activity score, and c-MYC activity score in PC patient cohorts. We applied the gene signature derived from our treatment of LNCaP cells with dilazep, as well as the previously published footprints of GATA2 (GSE63539 and He *et al.* 2014), AR (GSE63539 and He *et al.* 2014) and c-MYC (two signatures, one from overexpression of c-MYC for 12 hrs in LNCaP cells (GSE51384 and Barfeld *et al.* 2015) and the other from knockdown of c-MYC via siRNA in LNCaP (Koh *et al.* 2011)), and computed an activity score for each specimen in multiple previously reported human PC specimen cohorts: Taylor *et al.* (2010), Cai *et al.* (2013), and the Cancer Genome Atlas (TCGA) (https://doi.org/10.1530/ERC-21-0085.

Our HALLMARK results (Fig. 2B) highlighted that c-MYC signaling is blocked by dilazep. Moreover, ingenuity pathway analysis revealed that the top (most inhibited) upstream regulator in the dilazep signatures is the c-MYC pathway. In order to further validate these observations, we next compared our signatures of dilazep treatment of our three PC cell lines against several publicly available prostate-specific signatures of c-MYC activity: (1) following knockdown of c-MYC via siRNA in LNCaP, DU145, and PC3 PC cells (Koh *et al.* 2011), (2) upon overexpression of c-MYC for 5 or 12 h in LNCaP cells (GSE51384 and Barfeld *et al.* 2015), and (3) upon c-MYC overexpression in epithelial cells isolated from the mouse ventral prostate (GSE37428 and Ju *et al.* 2013). In all three of our PC cell lines, dilazep suppressed gene sets upregulated by c-MYC in prostate models and induced gene sets suppressed by c-MYC, highlighting that dilazep potently blocked the c-MYC program (Fig. 4A).

### The dilazep transcriptional program correlates with decreased GATA2 activity score, AR activity score, and Myc activity score in PC patient cohorts

We applied the gene signature derived from our treatment of LNCaP cells with dilazep, as well as the previously published footprints of GATA2 (GSE63539 and He *et al.* (2014)), AR (GSE63539 and He *et al.* (2014)) and c-MYC (two signatures, one from overexpression of c-MYC for 12 h in LNCaP cells (GSE51384 and Barfeld *et al.* (2015)) and the other from knockdown of c-MYC via siRNA in LNCaP Koh *et al.* (2011)), and computed an activity score for each specimen in multiple previously reported human PC specimen cohorts: Taylor *et al.* (2010), Cai *et al.* (2013), and the Cancer Genome Atlas (TCGA) (https://tcga-data.nci.nih.gov/tcga/). Within each human PC dataset, we found significant inverse correlation between dilazep scores and GATA2, AR, and c-MYC activity scores, providing further evidence that dilazep suppresses the GATA2, AR, and c-MYC programs (Fig. 4B).

### Dilazep suppresses protein expression of key cell-cycle regulators in androgen-dependent and CRPC cells

**Figure 5:**
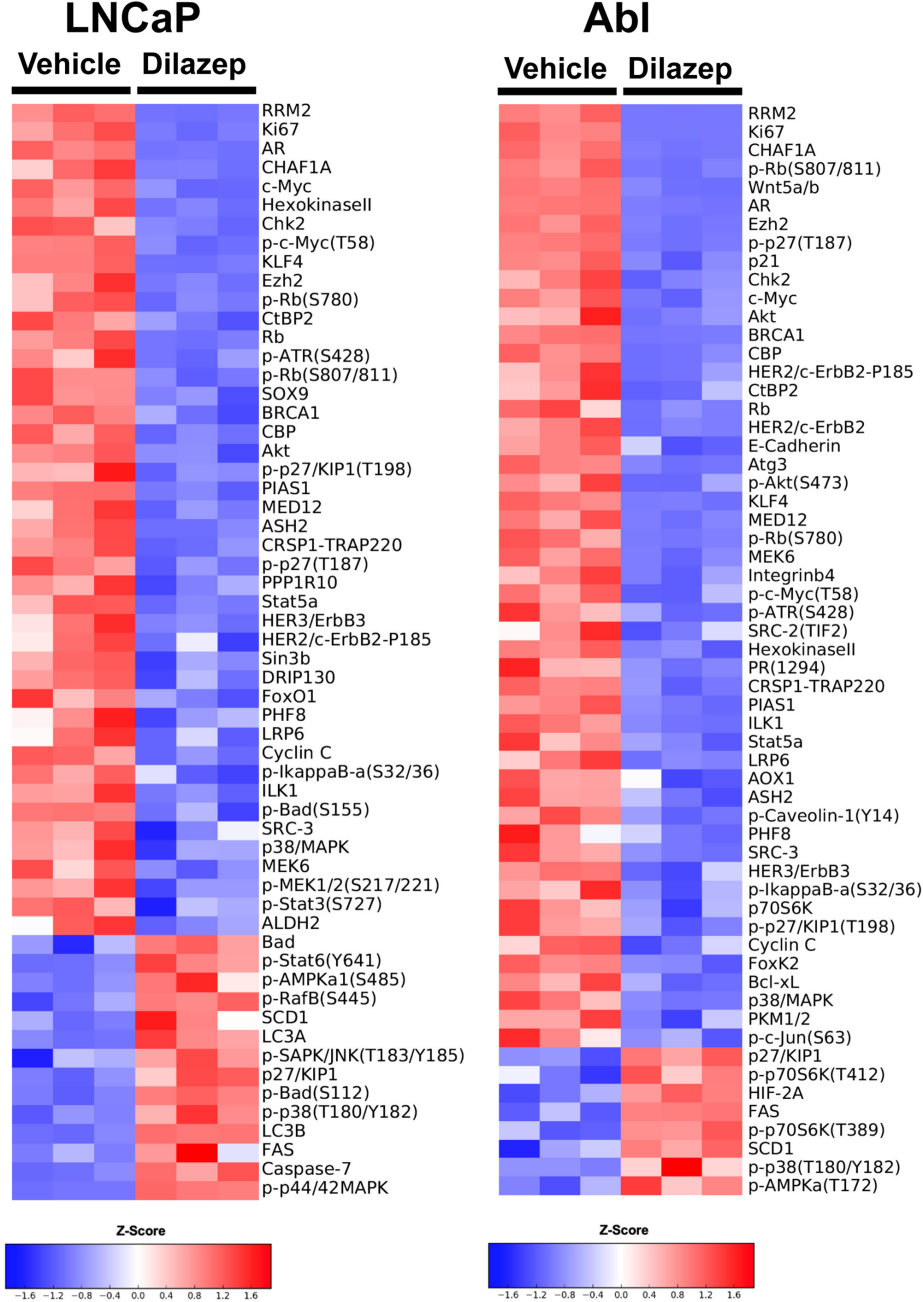
Dilazep treatment suppresses protein expression of AR, c-MYC, and other regulators of cell-cycle progression in LNCaP and Abl cells. The heatmap demonstrates the log_2_-based fold-change of protein expression, documented by RPPA analysis, for dilazep-treated cells compared to vehicle-treated cells. The proteomic signature caused by dilazep treatment was highly concordant between these two cell line models, as well as with LNCaP-MDVR cells (shown in Supplementary Fig. 7). A full color version of this figure is available at https://doi.org/10.1530/ERC-21-0085.

Having found transcriptional enrichments for cell cycle and c-MYC regulation in our dilazep-treated cells, we proceeded to protein level analysis using RPPA and immunoblotting. We assessed the effects of dilazep on LNCaP, Abl, and LNCaP-MDVR cells through RPPA. The proteomic signature caused by dilazep treatment was highly concordant between our three cell line models. The protein levels of AR and c-MYC were decreased across all three cell lines. We also found that dilazep suppressed the expression of several other PC drivers and cell-cycle regulators, including EZH2, RRM2, phospho-Rb, as well as several mediators of DNA damage repair such as BRCA1 and CHK2. Dilazep also suppressed, in at least one cell line, the protein expression of WNT5A, SOX9, AURKA, STAT5A, and the AR coactivators SRC-2 and SRC-3 (Fig. 5 and Supplementary Fig. 7).

**Figure 6:**
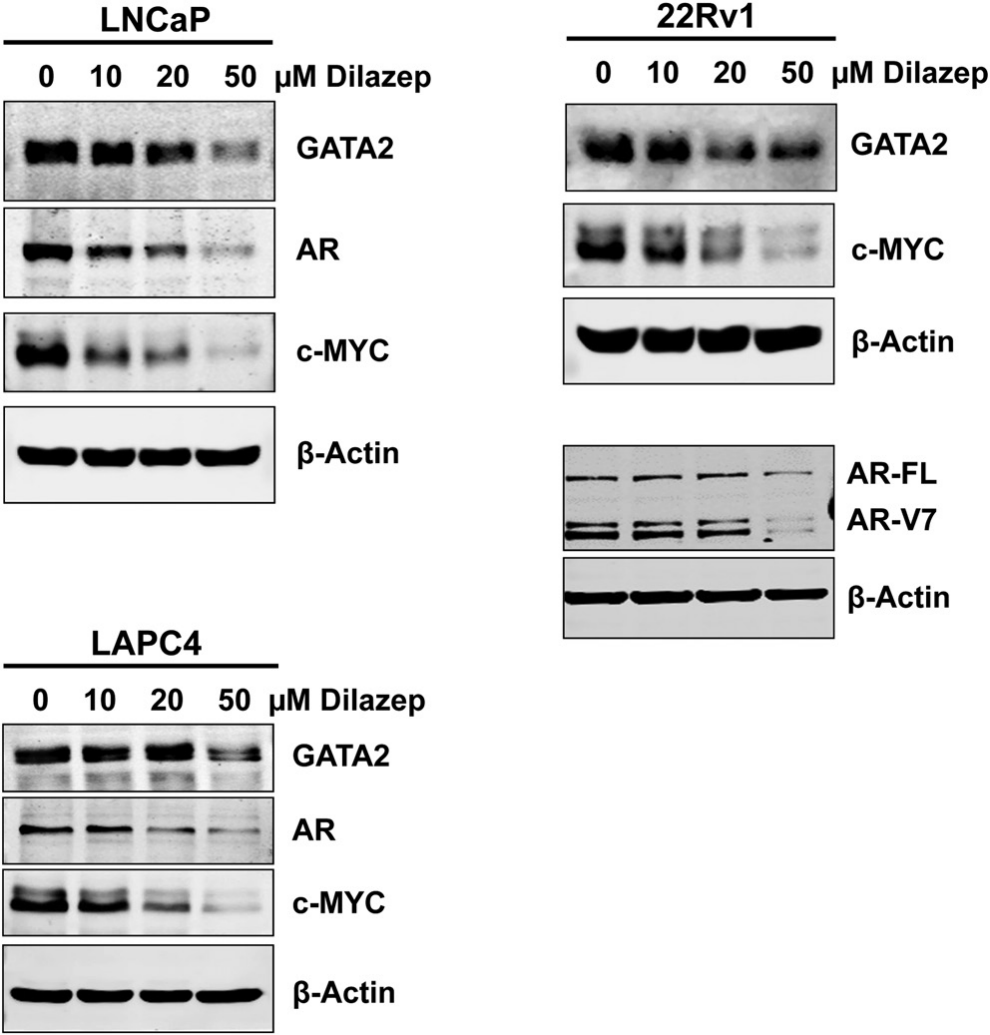
Immunoblotting for GATA2, AR, and c-MYC in LNCaP, 22Rv1, and LAPC4 cell treated with dilazep (0–50 µM) for 72 h.

To further expand these proteomic findings to additional cell line models, we treated 22Rv1 and LAPC4 cells with dilazep and assessed effects on protein expression of AR and c-MYC by immunoblotting (we also used LNCaP cells for continuity with the RPPA studies). Across all three cell lines, dilazep treatment dramatically decreased the protein levels of AR (including AR variants in 22Rv1 cells) and c-MYC in a dose-dependent manner (Fig. 6).

### GATA2 target engagement by dilazep

**Figure 7:**
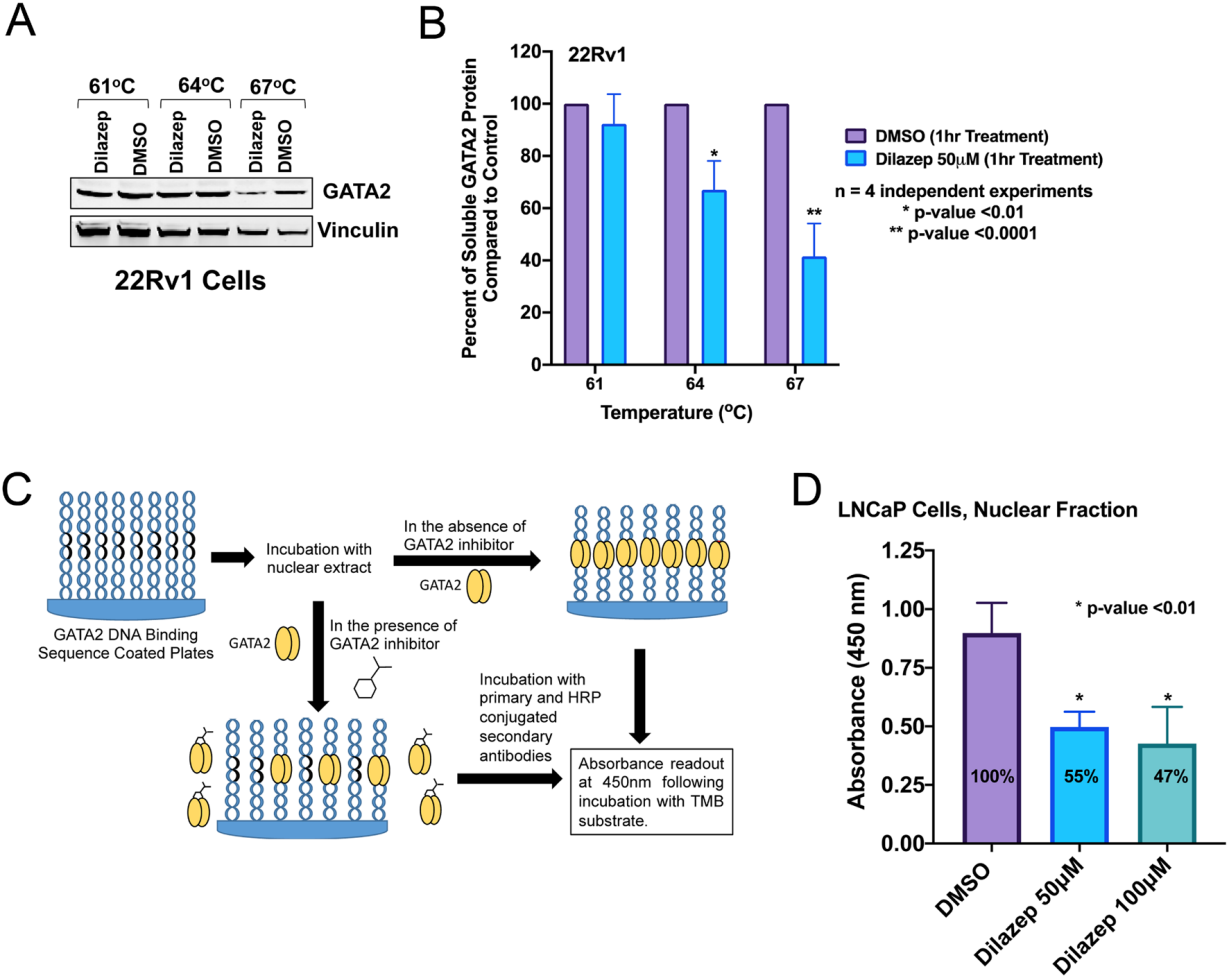
(A and B) Dilazep treatment resulted in destabilization of GATA2 protein. (A) Cellular thermal shift assay was performed in 22Rv1 cells following treatment with 50 μM of dilazep or DMSO for 1 h (described in ‘Materials and methods). Immunoblotting was carried out for GATA2. Vinculin served as control. A representative example (out of four independent experiments) is shown here. (B) Relative amounts of GATA2 to vinculin in dilazep-treated vs DMSO-treated samples were plotted from four independent experiments as the average ± s.d. These data support GATA2 target engagement by dilazep. (C and D) Dilazep inhibits GATA2 recruitment to DNA. (C) Schematic overview of the GATA2 DNA binding assay. (D) Nuclear extracts were prepared from LNCaP cells following treatment with DMSO (control) or dilazep (50 and 100 µM) for 16 h. DNA binding assay was performed with 50 µg of nuclear extract per well, as depicted above in C. GATA2 binding to its consensus DNA sequence was measured as described in Methods. Data from three independent experiments were plotted as the average ± s.d. All bar plots were generated using GraphPad Prism 8. A full color version of this figure is available at https://doi.org/10.1530/ERC-21-0085.

Following our gene expression studies, which demonstrated profound suppression of GATA2/AR transcriptional program by dilazep, we utilized cellular thermal shift assay (CETSA) to investigate the GATA2 target occupancy by dilazep. CETSA is a broadly applicable biophysical technique allowing detection of changes in ligand/drug binding interactions directly in intact cells. The technique is based on the thermal shift assay (TSA) concept where ligand binding affects protein stability. We treated PC cells with 50 μM of dilazep or DMSO for 1 h and obtained nuclear fraction from various conditions. Following a heating step, which causes protein denaturing and therefore precipitation, the remaining soluble fractions (i.e. folded proteins) were isolated and analyzed by immunoblotting. The levels of GATA2 were quantified and vinculin served as control. We observed that GATA2 became less stable following dilazep treatment (Tm = 64–67°C) compared to DMSO control in four independent experiments (Fig. 7A and B). To further confirm direct inhibition of GATA2 function by dilazep, we next performed a GATA2 DNA binding assay. Double-stranded oligonucleotides corresponding to GATA2 consensus DNA binding sequences were immobilized on plates and incubated with lysates from PC cells treated with DMSO or dilazep (Fig. 7C). Following dilazep treatment (50 or 100 μM of dilazep for 16 h), we observed significant reduction in GATA2 binding (Fig. 7D).

**Figure 8:**
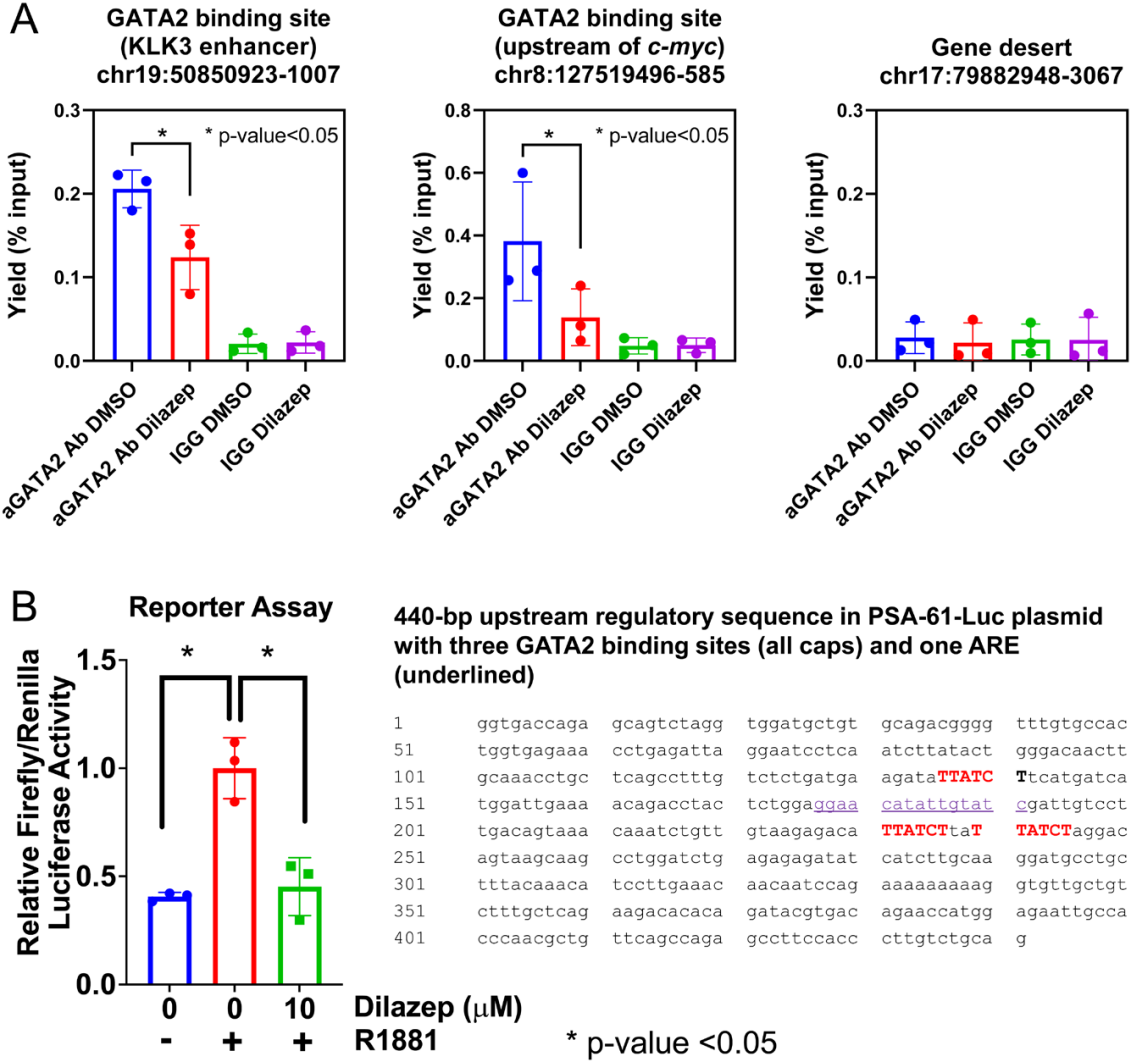
(A) Dilazep inhibits GATA2 recruitment to chromatin. LNCaP cells were treated with 10 µM of dilazep for 16 h and subjected to chromatin immunoprecipitation quantitative PCR (ChIP-qPCR) analysis for the presence of GATA2 on chromatin. Chromatin was fixed with formaldehyde, then DNA was extracted and sonicated into short fragments. GATA2-associated DNA was immunoprecipitated using anti-GATA2 or rabbit IgG as a negative control and analyzed by quantitative PCR as per Materials and methods. We selected two GATA2 binding sites based on prior GATA2 ChIP-seq profiles. The genomic coordinates for these sites are as indicated. Both sites harbor GATA2 binding sequences. Error bars indicate standard deviations for triplicates. (B) Dilazep suppresses transcriptional activity in a reporter assay based on the KLK3/PSA gene promoter/enhancer. 22Rv1 cells were transfected with reporter vector (pGL3/PSA61-Luc) and vectors encoding for Renilla luciferase (pGL4.75, Promega) and GATA2 (pcDNA3-GATA2) using jet PRIME transfection kit (Polyplus) according to the manufacturer’s protocol. The pGL3/PSA61-Luc harbors several GATA2 binding sites (those within a 440-bp regulatory sequence are shown here; for more details, please see Supplementary Text file) and three AREs (one of which is highlighted; two additional AREs are located right before the luciferase gene start site, please see Supplementary Text file). After 24 h, cells were treated with 5nM R1881 and either 10 µM dilazep or DMSO for 24 h. Luciferase activity in cells was measured with Dual-Luciferase Reporter Assay System (Promega) according to the manufacturer protocol. Relative firefly-to-Renilla luciferase activity from three independent experiments was plotted as the means ± s.d. All bar plots were generated using GraphPad Prism 8. A full color version of this figure is available at https://doi.org/10.1530/ERC-21-0085.

We also performed ChIP-qPCR in PC cells following dilazep treatment. We examined GATA2 binding at two genomic loci: one located near the KLK3/PSA gene and a second located near c-MYC. We observed significant reduction in GATA2 binding following dilazep treatment (Fig. 8A). Of note, treatment with dilazep at this concentration and duration does not lower GATA2 protein levels (Supplementary Fig. 8). To further examine the functional consequences of reduced GATA2 binding at the KLK3/PSA gene, we next utilized a reporter plasmid carrying a 6-kb fragment of the promoter/enhancer region of the PSA/KLK3 gene. This fragment harbors a 440-bp regulatory region (Cleutjens *et al.* 1997) which contains three GATA2 binding motifs and one ARE. Two additional AREs and several GATA2 binding motifs are also present on this plasmid further downstream, prior to the luciferase gene (Supplementary Text file). We observed significant reduction in transcriptional output as measured by luciferase activity following dilazep treatment (Fig. 8B). Together these series of experiments strongly indicate an on-target inhibition of GATA2 by dilazep.

### *In vivo* studies

**Figure 9:**
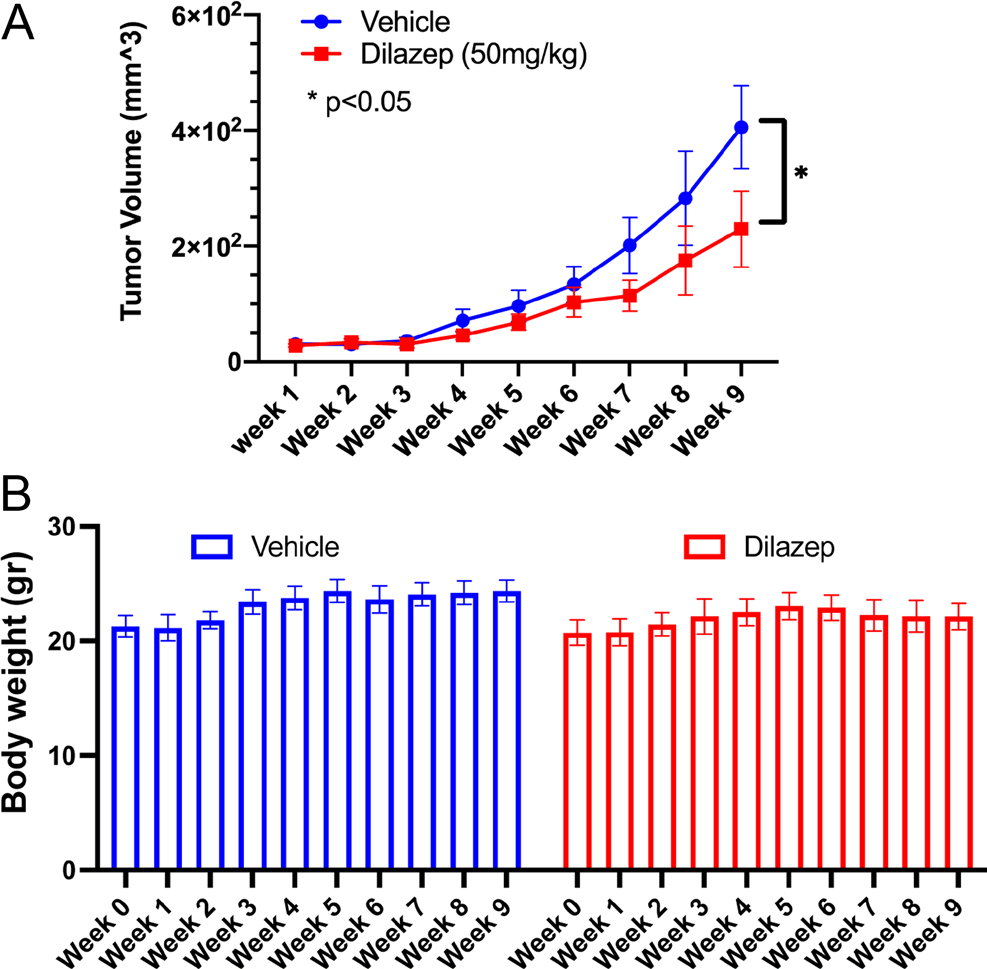
The CRPC PDX model, MDA-PCa-337A, generated by Dr Nora Navone at MD Anderson Cancer Center from a liver metastasis, was grown in castrated SCID male mice. Dilazep treatment, dissolved in molecular grade water at 200 mg/mL and administered intraperitoneally at 50 mg/kg daily 5 days per week (M–F), inhibited PDX growth (A, *P*  < 0.05 by two-way ANOVA), without causing loss of animal weight (B). Average values ± s.d. are shown. A full color version of this figure is available at https://doi.org/10.1530/ERC-21-0085.

The CRPC PDX model, MDA-PCa-337A, generated by Dr Nora Navone at MD Anderson Cancer Center from a liver metastasis, was grown in castrated SCID male mice. Dilazep, dissolved in molecular grade water at 200 mg/mL and administered intraperitoneally at 50 mg/kg daily 5 days per week (M–F), inhibited PDX growth (Fig. 9A), without causing loss of animal weight (Fig. 9B).

## Discussion

Novel therapeutic targets are needed in CRPC, in order to improve patient outcomes. GATA2 is an important PC and CRPC driver (Perez-Stable *et al.* 2000, Bohm *et al.* 2009, Chiang *et al.* 2014, He *et al.* 2014, Wu *et al.* 2014, Vidal *et al.* 2015, Chaytor *et al.* 2019). However, pharmacologic targeting of GATA2 is hindered by the lack of a ligand-binding pocket or an actionable site of protein activation and by the absence of an established 3-dimensional structure for the full-length GATA2.

In the present study, we predicted bioinformatically the vasodilator dilazep to be a GATA2 inhibitor. We proceeded to validate the effect of dilazep on GATA2 using CETSA (an indirect assay of target engagement) and also found that dilazep inhibits GATA2 recruitment to chromatin and DNA (using ChIP-qPCR and a GATA2 DNA-binding assay, respectively) and activity in a reporter assay. Importantly, dilazep exerted anticancer activity against GATA2-dependent (both androgen-dependent and androgen-independent) PC cell lines, while its effect on proliferation of PC-3 and RWPE-1 cells was minimal and only at high concentration. The latter finding is in agreement with our prior report that the AR-negative PC-3 cells and benign prostate cell models such as the RWPE-1 cells are resistant to GATA2 targeting via siRNA and a different small molecule inhibitor (He *et al.* 2014). Dilazep was also active against a CRPC PDX model *in vivo*, without causing mouse weight loss or apparent toxicity. Using protein and RNA profiling, we established that dilazep suppresses transcriptional programs associated with cell cycle, mitosis, DNA replication, DNA repair, E2F, and c-MYC targets in PC cells (including CRPC cells). Several cell regulators were suppressed concordantly across our cell line panel, including Ki67, p-Rb(S807/811), cyclins, and members of the CENP family.

In addition, our global gene expression profiling and confirmatory RT-qPCR studies established, for the first time, that a small molecule can suppress the GATA2 transcriptional profile. GSEA revealed that the transcriptional footprint of dilazep strongly matched that caused by GATA2 siRNA profile in all three PC cell lines, both as far as the upregulated and the downregulated genes. Application of the dilazep signature to patient samples also showed that it is associated with decreased activity of GATA2. Moreover, GSEA demonstrated that dilazep strongly matched the transcriptional footprint caused by AR siRNA in both androgen-dependent LNCaP cells and androgen-independent Abl cells. In further exploration of this effect, we found that dilazep suppressed expression of AR itself across a wide panel of PC cell lines, including the androgen-dependent LNCaP and LAPC4 cells, the androgen-independent Abl, the enzalutamide-resistant MDVR, and the AR variant(+) 22Rv1 cells. This validates our hypothesis that pharmacological targeting of GATA2 can serve as a surrogate mechanism to inhibit AR, which is of particular importance in CRPC expressing AR variants that are resistant to enzalutamide and abiraterone.

Moreover, our GSEA revealed, using a wide variety of c-MYC-driven signatures, that c-MYC signaling was a target of dilazep in all three PC cell lines. In agreement, we found very potent suppression of c-MYC mRNA (Supplementary Fig. 5) and protein expression (Fig. 5 and Supplementary Figs 6, 7) by dilazep in all PC cell lines tested. Application of the dilazep signature to patient samples also showed that it is associated with decreased transcriptional activity of c-MYC. Collectively, our findings suggests that c-MYC expression and function are highly GATA2-dependent in PC cells, and extremely sensitive to GATA2 inhibition, thus providing an opportunity to effectively silence the expression of an otherwise undruggable major driver of PC (Buttyan *et al.* 1987, Bernard *et al.* 2003, Edwards *et al.* 2003, Ellwood-Yen *et al.* 2003).

Dilazep also suppressed the expression of several other well-known PC drivers (Fig. 5 and Supplementary Figs 5, 7), including FOXM1 (Aytes *et al.* 2014, Lin *et al.* 2016), CENPF (Aytes *et al.* 2014, Lin *et al.* 2016), EZH2 (Varambally *et al.* 2002, Bryant *et al.* 2007, Xu *et al.* 2012, Dardenne *et al.* 2016), UBE2C (Wang *et al.* 2009, 2011, Chen *et al.* 2011), RRM2 (Mazzu *et al.* 2019), as well as several mediators of DNA damage repair such as BRCA1 and CHK2. Dilazep also suppressed, in at least one cell line, the AR coactivators SRC-2 and SRC-3 and the PC drivers WNT5A/B, SOX9, AURKA, and STAT5A. Moreover, dilazep suppressed genes of the *NANOG, SOX2,* and *OCT4* stem cell transcriptional programs, which are known to play important roles in PC (Gu *et al.* 2007, Sotomayor *et al.* 2009, Jeter *et al.* 2011, 2016, Chu *et al.* 2014, Kregel *et al.* 2014, Jiang *et al.* 2016, Hepburn *et al.* 2019, Sanchez *et al.* 2020, Zadvornyi *et al.* 2020).

Having discovered this capacity of dilazep to suppress several well-known PC drivers, we propose that GATA2 inhibition would be expected to affect several hallmarks of cancer. In agreement, the transcriptional program triggered by dilazep was associated with suppression of metastasis-associated genes (Fig. 3B). This supports that dilazep suppresses the metastatic program of PC cells and is in agreement with our prior findings that GATA2 expression is higher in metastatic PC compared to primary PC human specimens and also is associated with higher risk of recurrence after prostatectomy (He *et al.* 2014).

In conclusion, our study provides proof of principle that a small molecule can inhibit the GATA2 and c-MYC transcriptional programs in PC cells and can exert anticancer effects *in vitro* and *in vivo*. The ability of a GATA2 inhibitor to suppress several AR-mediated and AR-independent PC driver pathways and to overcome resistance to hormonal therapy opens new treatment opportunities for CRPC patients.

## Supplementary Material

Suppl. Figure 1. Using the prediction algorithm SuperPred we identified dilazep, a vasodilator, as a clinically available drug with potential inhibitory activity against GATA-2

Suppl. Figure 2. Heatmap representation of protein expression changes (log2 scale) revealed by RPPA analysis to be differentially expressed in enzalutamide-resistant LNCaP-MDVR cells compared to parental LNCaP cells (P<0.05 & FC>1.25 or <1/1.25)

Suppl. Fig. 3 A. GATA2 siRNA inhibits growth of MDVR cells (MTT assay performed 96 hrs after siRNA transfection), similarly to its previously reported effect in LNCaP and Abl cells. siNT: Non-Target siRNA. Data are shown as average ± SD. B. Dilazep inhibits proliferation of MDVR PC cells: MTT assay after treatment with increasing concentrations of dilazep (0-50 µM) for 96 hrs. O.D. was calculated as absorbance at 570 nM – absorbance at 630 nM and normalized to the respective vehicle controls. Data are shown as average ± SD. C. The GATA2-independent, AR-independent PC3 and RWPE-1 cells were significantly less sensitive to dilazep and only at high concentration: MTT assay after treatment with increasing concentrations of dilazep (0-50 µM) for 96 hrs. O.D. was calculated as absorbance at 570 nM– absorbance at 630 nM and normalized to the respective vehicle controls. Data are shown as average ± SD.

Suppl. Fig. 3 D-G. EdU proliferation assay in 22Rv1 cells (D, E) and LNCaP cells (F, G) treated with dilazep (0, 20, 50 µM) for 24h. Representative flow cytometry dot plots (D, F) obtained by a double labelling of the cells with EdU and DAPI. EdU incorporation into DNA (90 minute labelling with 10 µM EdU) is displayed on the ordinate and DNA content (DAPI) is shown on the abscissa. The cells in the green S square correspond to EdU labelled cells (S phase of the cell cycle). The cells in G1 and G2 squares represent cells with a 2C (G1 phase of the cell cycle) and 4C (G2 phase of the cell cycle) DNA content respectively that did not incorporate EdU. The cells in subG1 square represent cells with a DNA content less than 2C (apoptotic cells). Bar charts (E, G) show a summary of S-phase frequency in cells grown in the presence of dilazep in independent experiments.

Suppl. Fig. 4A Dilazep suppresses DNA repair signaling in PC cells. Using GSEA, we analyzed our dilazep signatures, across all three PC cell lines tested, and compared them with the Molecular Signature Database (MSigDB). We found significant suppression of genesets related to DNA repair. All P<0.001.

Suppl. Fig. 4B Dilazep suppresses stemness-related signaling in PC cells. Using GSEA, we analyzed our dilazep signatures, across all three PC cell lines tested, and compared them with the Molecular Signature Database (MSigDB). We found significant suppression of genesets related to embryonic stem (ES) cells and stemness. All P<0.001.

Suppl. Figure 5A. Expression changes (log<sub>2</sub>FC) in selected mRNAs revealed by gene expression profiling analysis, after treatment with dilazep (50 μM for 48 hrs) in parental LNCaP, (LNCaP)-Abl and (LNCaP)-MDVR cells (adjusted P<0.05). The transcriptional signature caused by dilazep treatment was highly concordant between our three cell line models. The last column highlights genes whose expression was suppressed by GATA2 siRNA in LNCaP cells.

Suppl. Figure 5B. Quantitative real-time RT-PCR results for selected mRNAs (AURKA, CCNA2, CENPF, MYC, RAD51AP1) in (LNCaP)-Abl and (LNCaP)-MDVR cells treated with 50 μM dilazep for 48 hrs. T-test was used for statistical analysis (significance set at P<0.05).

Suppl. Fig. 6 Dilazep suppresses AR-driven signaling in PC cells. We compared, via GSEA, the transcriptional footprint of dilazep with that of AR siRNA in androgen-dependent LNCaP and androgen-independent Abl cells. In both cell lines, genes downregulated by AR siRNA were strongly enriched among the genes suppressed by dilazep, while genes upregulated by AR siRNA were strongly enriched among the genes induced by dilazep in the same cell line. This provides evidence that dilazep indeed suppresses the AR axis, not only under androgen-dependent conditions but also in CRPC cells.

Suppl. Fig. 7 Dilazep treatment diminishes protein expression of AR, c-Myc and regulators of cell-cycle progression. Heatmap representation of selected proteins revealed to have been changed via RPPA analysis. LNCaP-MDVR cells were treated with 50 µM of dilazep (or H2O control) for 48 hrs and submitted in triplicate for RPPA analysis. Heatmap reveals protein expression change, in log(2) scale, for dilazep treated cells compared to vehicle treated cells. The proteomic signature caused by dilazep treatment in MDVR cells was highly concordant with that seen in LNCaP and Abl cells (Fig. 5).

Suppl. Fig. 8 Dilazep treatment in LNCaP cells for 16 hrs does not suppress total GATA2 protein levels.

Supplementary Materials

Suppl Table 1: Antibodies used for RPPA

## Declaration of interest

The authors declare that there is no conflict of interest that could be perceived as prejudicing the impartiality of the research reported.

## Funding

This work was also supported by the American Cancer Society http://dx.doi.org/10.13039/100000048 RSG-14-218-01-TBG (to N M), the Prostate Cancer Foundation http://dx.doi.org/10.13039/100000892 (S K, C C and N M), NIH 5T32CA174647-03 (S K), Department of Defense Congressionally Directed Medical Research Programs http://dx.doi.org/10.13039/100000090/Prostate Cancer Research Program Idea Development Awards W81XWH-15-1-0674, W81XWH-17-1-0298 and W81XWH-18-1-0288, National Cancer Institute http://dx.doi.org/10.13039/100000054 Grant U54-CA233223 (S K and N M), the Cancer Prevention & Research Institute of Texas (CPRIT) award RP150648, the Terry Fox Foundation http://dx.doi.org/10.13039/501100002655, the Sidney Kimmel Foundation http://dx.doi.org/10.13039/100011034, the Elsa U. Pardee Foundation and SPORE P50CA58183 and P50CA186784 (N M). The authors also would like to acknowledge the assistance of the Dan L. Duncan Cancer Center Shared Resources, in particular the Antibody-based Proteomics Core/Shared Resource (supported by the NCI Cancer Center Support Grant P30CA125123). This work was also supported in part by NIH S10 instrument award (S10OD028648, SH), CPRIT Proteomics & Metabolomics Core Facility Support Award (RP170005 & RP210227) (K R, S H, C C), CPRIT Award RP150648, RP200504 (CPRIT Epigenomic Core), and NIEHS grants 1P30ES030285 and 1P42ES027725. We thank Ms Fuli Jia and Dr Danli Wu from the Antibody-based Proteomics Core/Shared Resource for their excellent technical assistant in performing RPPA experiments. This project was supported by the Cytometry and Cell Sorting Core at Baylor College of Medicine http://dx.doi.org/10.13039/100007915 with funding from the CPRIT Core Facility Support Award (CPRIT-RP180672), the NIH (P30 CA125123 and S10 RR024574) and the expert assistance of Joel M Sederstrom.
