# Supplementary material for: Inhibition of GATA2 in prostate cancer by a clinically available small molecule
Source: Endocr Relat Cancer. 2021 Oct 12;29(1):15–31. doi: 10.1530/ERC-21-0085 (PMC8634153; doi:10.1530/ERC-21-0085)
Supplement: Suppl. Figure 1. Using the prediction algorithm SuperPred we identified dilazep, a vasodilator, as a clinically available drug with potential inhibitory activity against GATA-2 [file supplementary_figure_1.pdf]

Suppl. Figure 1. Using the prediction algorithm SuperPred we identified dilazep, a vasodilator, as a clinically available drug with potential inhibitory activity against GATA-2

Input structure: K-7174

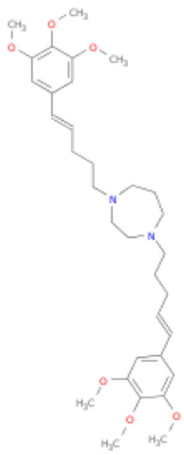

Most similar drug

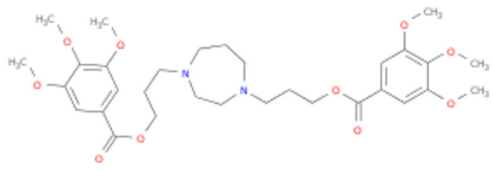

Information input structure

Name	1,4-bis[(4E)-5-(3,4,5-trimethoxyphenyl)pent-4-en-1-yl]-1,4-diazepane
Formula	$C_{33}H_{48}N_2O_6$
Molweight	568.744
xlogP	5.909
Heavy Atoms	41
Rotatable Bonds	16
H-bond Donors	0
H-bond Acceptors	8
Bonds	43
Rings	3
Polar Surface Area	61.860
Total Charge	0

Drug properties

Name	Dilazep
Formula	$C_{31}H_{44}N_2O_{10}$
Molweight	604.688
logP	3.416
Heavy Atoms	43
Rotatable Bonds	18
H-bond Donors	0
H-bond Acceptors	12
Bonds	45
Rings	3
Polar Surface Area	114.46
Total Charge	0
