# Supplementary material for: Inhibition of GATA2 in prostate cancer by a clinically available small molecule
Source: Endocr Relat Cancer. 2021 Oct 12;29(1):15–31. doi: 10.1530/ERC-21-0085 (PMC8634153; doi:10.1530/ERC-21-0085)

Suppl. Figure 2. Heatmap representation of protein expression changes (log_2 scale) revealed by RPPA analysis to be differentially expressed in enzalutamide-resistant LNCaP-MDVR cells compared to parental LNCaP cells (P<0.05 & FC>1.25 or <1/1.25)

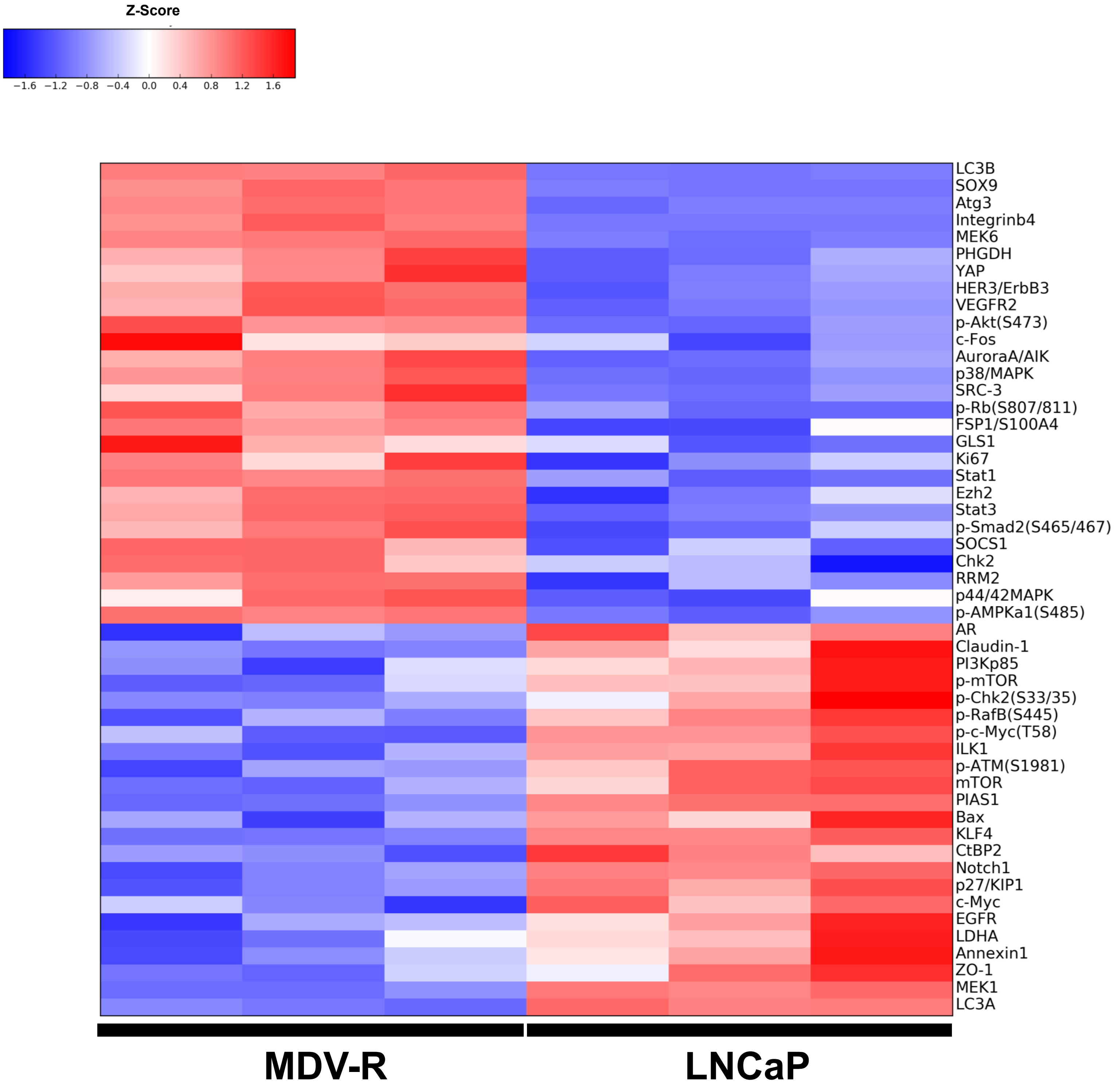

Supplement: Suppl. Figure 2. Heatmap representation of protein expression changes (log2 scale) revealed by RPPA analysis to be differentially expressed in enzalutamide-resistant LNCaP-MDVR cells compared to parental LNCaP cells (P<0.05 & FC>1.25 or <1/1.25) [file supplementary_figure_2.pdf]
