# Supplementary material for: Inhibition of GATA2 in prostate cancer by a clinically available small molecule
Source: Endocr Relat Cancer. 2021 Oct 12;29(1):15–31. doi: 10.1530/ERC-21-0085 (PMC8634153; doi:10.1530/ERC-21-0085)

Suppl. Fig. 3

A. GATA2 siRNA inhibits growth of MDVR cells (MTT assay performed 96 hrs after siRNA transfection), similarly to its previously reported effect in LNCaP and Abl cells.
siNT: Non-Target siRNA. Data are shown as average ± SD.

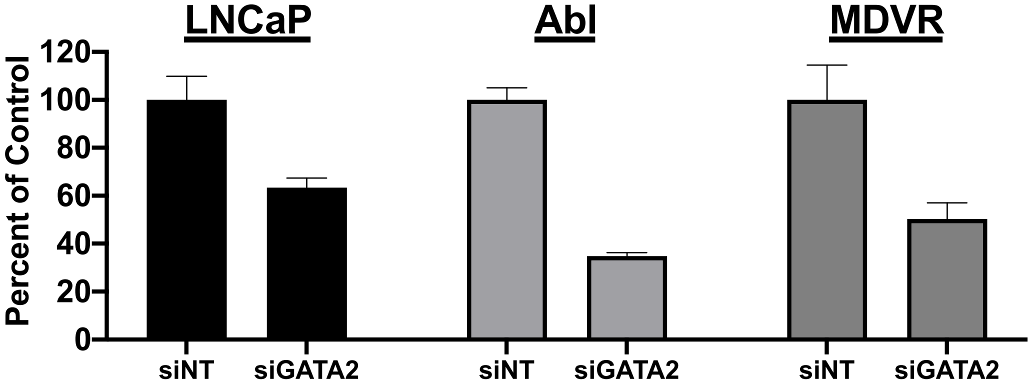

B. Dilazep inhibits proliferation of MDVR PC cells: MTT assay after treatment with increasing concentrations of dilazep (0-50 μM) for 96 hrs. O.D. was calculated as absorbance at 570 nM – absorbance at 630 nM and normalized to the respective vehicle controls. Data are shown as average ± SD.

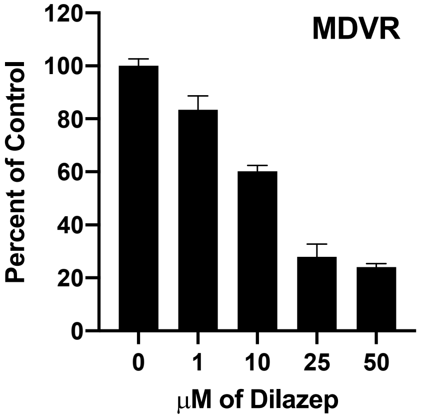

C. The GATA2-independent, AR-independent PC3 and RWPE-1 cells were significantly less sensitive to dilazep and only at high concentration: MTT assay after treatment with increasing concentrations of dilazep (0-50 μM) for 96 hrs. O.D. was calculated as absorbance at 570 nM – absorbance at 630 nM and normalized to the respective vehicle controls. Data are shown as average ± SD.

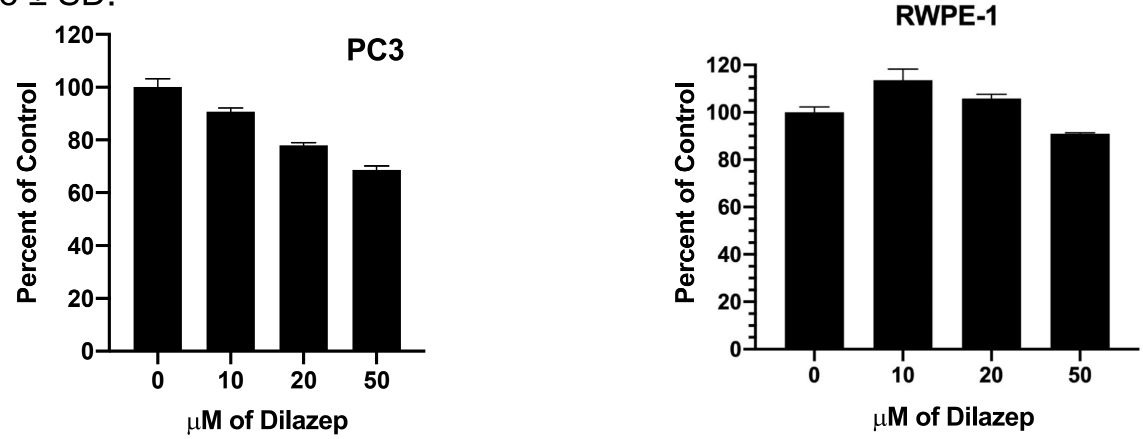

Supplement: Suppl. Fig. 3 A. GATA2 siRNA inhibits growth of MDVR cells (MTT assay performed 96 hrs after siRNA transfection), similarly to its previously reported effect in LNCaP and Abl cells. siNT: Non-Target siRNA. Data are shown as average ± SD. B. Dilazep inhibits proliferation of MDVR PC cells: MTT assay  [file supplementary_figure_3.pdf]
