# Supplementary material for: Inhibition of GATA2 in prostate cancer by a clinically available small molecule
Source: Endocr Relat Cancer. 2021 Oct 12;29(1):15–31. doi: 10.1530/ERC-21-0085 (PMC8634153; doi:10.1530/ERC-21-0085)

Suppl. Fig. 3

D-G. EdU proliferation assay in 22Rv1 cells (D, E) and LNCaP cells (F, G) treated with dilazep (0, 20, 50 μM) for 24h. Representative flow cytometry dot plots (D, F) obtained by a double labelling of the cells with EdU and DAPI. EdU incorporation into DNA (90 minute labelling with 10 μM EdU) is displayed on the ordinate and DNA content (DAPI) is shown on the abscissa. The cells in the green S square correspond to EdU labelled cells (S phase of the cell cycle). The cells in G1 and G2 squares represent cells with a 2C (G1 phase of the cell cycle) and 4C (G2 phase of the cell cycle) DNA content respectively that did not incorporate EdU. The cells in subG1 square represent cells with a DNA content less than 2C (apoptotic cells). Bar charts (E, G) show a summary of S-phase frequency in cells grown in the presence of dilazep in independent experiments.

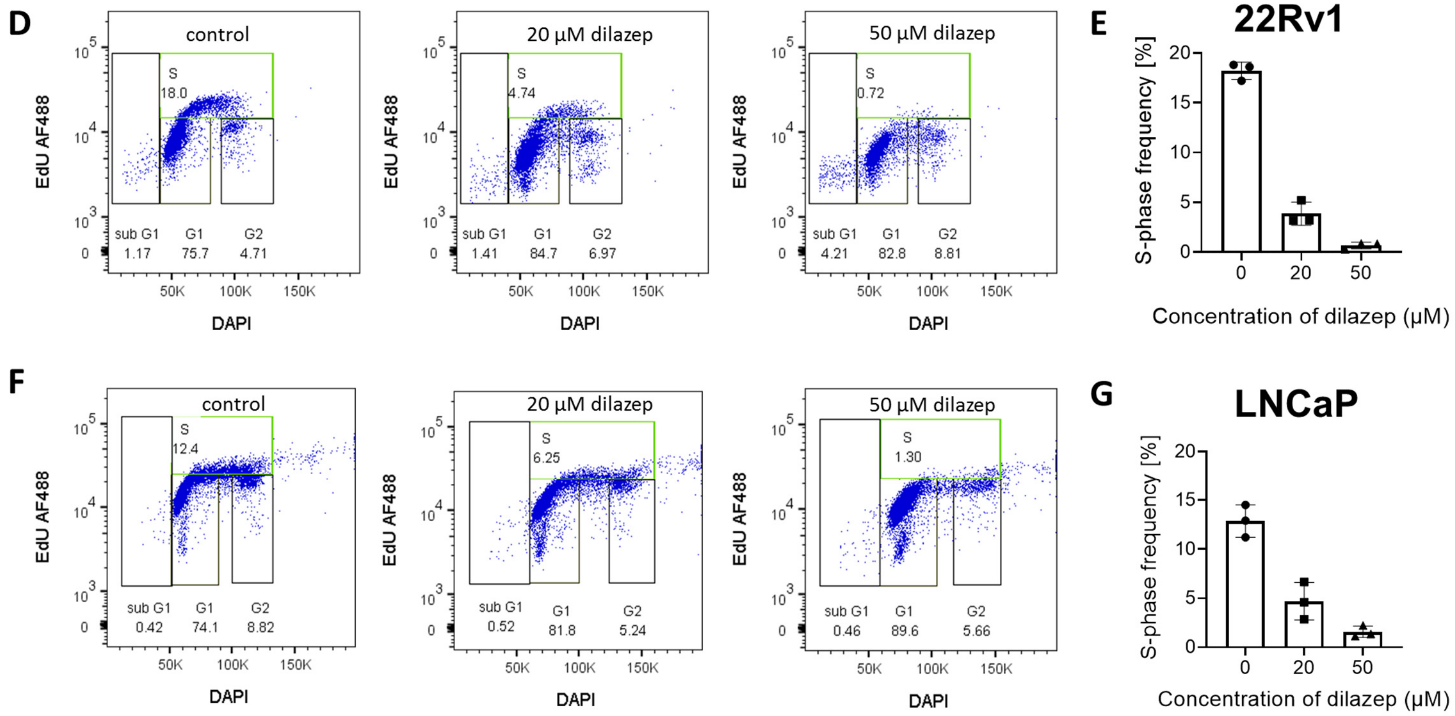

Supplement: Suppl. Fig. 3 D-G. EdU proliferation assay in 22Rv1 cells (D, E) and LNCaP cells (F, G) treated with dilazep (0, 20, 50 µM) for 24h. Representative flow cytometry dot plots (D, F) obtained by a double labelling of the cells with EdU and DAPI. EdU incorporation into DNA (90 minute labelling with 10 µ [file supplementary_figure_4.pdf]
