# Supplementary material for: Inhibition of GATA2 in prostate cancer by a clinically available small molecule
Source: Endocr Relat Cancer. 2021 Oct 12;29(1):15–31. doi: 10.1530/ERC-21-0085 (PMC8634153; doi:10.1530/ERC-21-0085)

Suppl. Fig. 4A

Dilazep suppresses DNA repair signaling in PC cells. Using GSEA, we analyzed our dilazep signatures, across all three PC cell lines tested, and compared them with the Molecular Signature Database (MSigDB). We found significant suppression of genesets related to DNA repair.
All P<0.001.

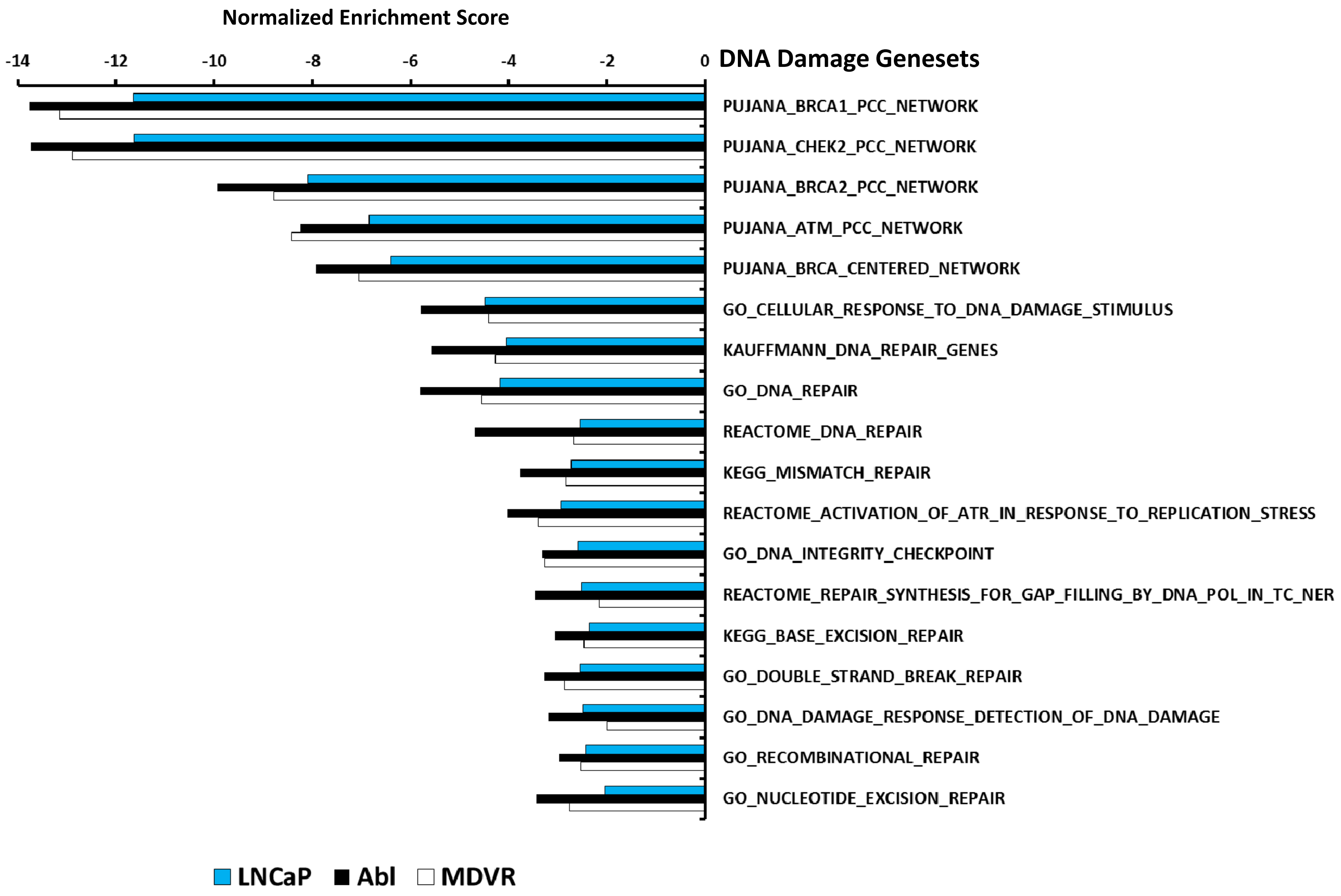

Supplement: Suppl. Fig. 4A Dilazep suppresses DNA repair signaling in PC cells. Using GSEA, we analyzed our dilazep signatures, across all three PC cell lines tested, and compared them with the Molecular Signature Database (MSigDB). We found significant suppression of genesets related to DNA repair. All P<0.001 [file supplementary_figure_5.pdf]
