# Supplementary material for: Inhibition of GATA2 in prostate cancer by a clinically available small molecule
Source: Endocr Relat Cancer. 2021 Oct 12;29(1):15–31. doi: 10.1530/ERC-21-0085 (PMC8634153; doi:10.1530/ERC-21-0085)
Supplement: Suppl. Figure 5A. Expression changes (log2FC) in selected mRNAs revealed by gene expression profiling analysis, after treatment with dilazep (50 μM for 48 hrs) in parental LNCaP, (LNCaP)-Abl and (LNCaP)-MDVR cells (adjusted P<0.05). The transcriptional signature caused by dilazep treatment was highl [file supplementary_figure_7.pdf]

**Suppl. Figure 5A.** Expression changes ($log_2FC$) in selected mRNAs revealed by gene expression profiling analysis, after treatment with dilazep (50 µM for 48 hrs) in parental LNCaP, (LNCaP)-Abl and (LNCaP)-MDVR cells (adjusted P<0.05). The transcriptional signature caused by dilazep treatment was highly concordant between our three cell line models. The last column highlights genes whose expression was suppressed by GATA2 siRNA in LNCaP cells.

| Gene | LNCaP | Abl | MDVR | Function | Suppressed by GATA2 siRNA |
|---|---|---|---|---|---|
| KLK3 | -0.612 | N/A | | Canonical AR target gene | Yes |
| HK2 | -0.472 | | | Canonical AR target gene | Yes |
| INPP4B | -0.548 | | | Canonical AR target gene | Yes |
| CCNA2 | -1.476 | -1.718 | -1.047 | Cell cycle regulator | Yes |
| CDC20 | -0.869 | -2.413 | -1.153 | Cell cycle regulator | Yes |
| CEP55 | -1.047 | -2.226 | -0.887 | Cell cycle regulator | Yes |
| CENPF | -0.661 | -1.704 | -1.111 | Cell cycle regulator | Yes |
| CCNB2 | -1.37 | -2.655 | -1.376 | Cell cycle regulator | Yes |
| NUSAP1 | -1.671 | -2.962 | -1.091 | Cell cycle regulator | Yes |
| CENPE | -0.994 | -1.242 | -1.022 | Cell cycle regulator | Yes |
| AURKB | -0.571 | -2.055 | -0.756 | Cell cycle regulator | Yes |
| FOXM1 | -0.858 | -1.676 | -0.721 | Cell cycle regulator | Yes |
| AURKA | -1.024 | -2.174 | -1.26 | Cell cycle regulator | Yes |
| UHRF1 | -1.218 | -2.232 | -0.434 | Cell cycle regulator | Yes |
| MYC | -1.08 | -1.73 | -0.586 | Cell cycle regulator | Yes |
| CCNE2 | -0.815 | -1.536 | NS | Cell cycle regulator | Yes |
| SKP2 | -0.834 | -1.115 | -0.443 | Cell cycle regulator | Yes |
| NEK2 | -1.355 | -2.989 | -1.306 | Cell cycle regulator | Yes |
| UBE2C | -1.621 | -3.225 | -1.46 | Cell cycle regulator | Yes |
| EZH2 | -0.386 | -0.738 | -0.448 | Cell cycle regulator | Yes |
| DLGAP5 | -1.603 | -2.014 | -1.293 | Cell cycle regulator | Yes |
| TM4SF1 | -1.82 | -1.485 | -1.17 | Cell cycle regulator | Yes |
| ATAD2 | -1.05 | -1.849 | -0.792 | Cell cycle regulator | Yes |
| CDCA2 | NS | -0.78 | -0.512 | Cell cycle regulator | Yes |
| CCNB1 | -0.999 | -1.805 | -0.984 | Cell cycle regulator | Yes |
| CENPM | -0.635 | -2.023 | -0.613 | Cell cycle regulator | Yes |
| CENPA | -0.887 | -1.362 | -0.707 | Cell cycle regulator | Yes |
| CDCA3 | -0.905 | -2.244 | -1.043 | Cell cycle regulator | Yes |
| CDC25C | -0.482 | -0.901 | -0.657 | Cell cycle regulator | Yes |
| CENPH | -0.937 | -1.192 | -0.623 | Cell cycle regulator | No |
| BIRC5 | -0.507 | -2.133 | -0.96 | Cell survival | Yes |
| TOP2A | -1.171 | -2.449 | -1.207 | DNA Replication, Recombination, and Repair | Yes |
| FANCI | -0.805 | -2.184 | -0.525 | DNA Replication, Recombination, and Repair | Yes |
| BRCA1 | NS | -1.467 | -0.411 | DNA Replication, Recombination, and Repair | Yes |
| CHEK2 | -0.807 | -1.341 | -0.453 | DNA Replication, Recombination, and Repair | Yes |
| MDC1 | -0.451 | -1.874 | -0.766 | DNA Replication, Recombination, and Repair | Yes |
| RRM2 | -0.435 | -0.799 | -0.364 | DNA Replication, Recombination, and Repair | Yes |
| RAD51AP1 | -1.284 | -2.399 | -1.087 | DNA Replication, Recombination, and Repair | Yes |
| KIF4A | -0.495 | -1.027 | -0.698 | DNA Replication, Recombination, and Repair | Yes |
| SGOL1 | -0.56 | -0.899 | -0.35 | DNA Replication, Recombination, and Repair | Yes |
| CDCA8 | -0.463 | -1.236 | -0.724 | DNA Replication, Recombination, and Repair | Yes |
| NCAPG2 | -0.553 | -0.727 | -0.52 | DNA Replication, Recombination, and Repair | Yes |
| FANCD2 | -0.95 | -1.958 | -0.621 | DNA Replication, Recombination, and Repair | Yes |
| CHEK1 | -0.707 | -1.289 | -0.486 | DNA Replication, Recombination, and Repair | Yes |
| FEN1 | -0.656 | -2.381 | -0.666 | DNA Replication, Recombination, and Repair | Yes |
| CSE1L | -0.404 | -2.032 | -0.587 | DNA Replication, Recombination, and Repair | Yes |
| PARP2 | -0.596 | -1.076 | -0.484 | DNA Replication, Recombination, and Repair | Yes |
| ALDH1B1 | -0.403 | -0.872 | -0.428 | Metabolism | Yes |
