# Supplementary material for: Inhibition of GATA2 in prostate cancer by a clinically available small molecule
Source: Endocr Relat Cancer. 2021 Oct 12;29(1):15–31. doi: 10.1530/ERC-21-0085 (PMC8634153; doi:10.1530/ERC-21-0085)

Suppl. Figure 5B. Quantitative real-time RT-PCR results for selected mRNAs (AURKA, CCNA2, CENPF, MYC, RAD51AP1) in (LNCaP)-Abl and (LNCaP)-MDVR cells treated with 50 μM dilazep for 48 hrs. T-test was used for statistical analysis (significance set at $P<0.05$).

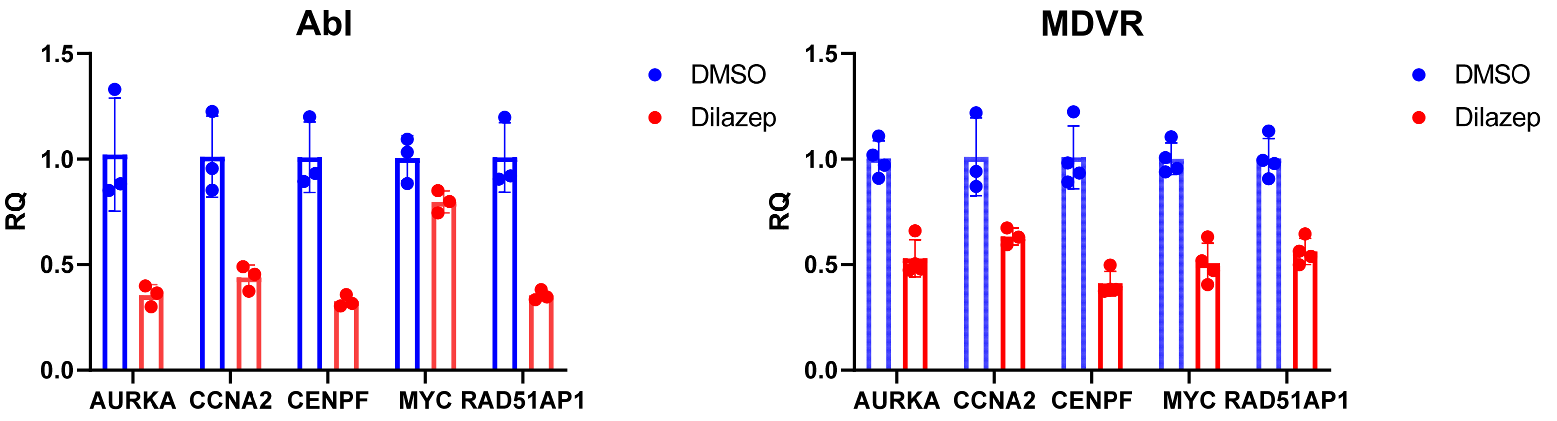

Supplement: Suppl. Figure 5B. Quantitative real-time RT-PCR results for selected mRNAs (AURKA, CCNA2, CENPF, MYC, RAD51AP1) in (LNCaP)-Abl and (LNCaP)-MDVR cells treated with 50 μM dilazep for 48 hrs. T-test was used for statistical analysis (significance set at P<0.05). [file supplementary_figure_8.pdf]
