# Supplementary material for: Inhibition of GATA2 in prostate cancer by a clinically available small molecule
Source: Endocr Relat Cancer. 2021 Oct 12;29(1):15–31. doi: 10.1530/ERC-21-0085 (PMC8634153; doi:10.1530/ERC-21-0085)

Suppl. Fig. 6

Dilazep suppresses AR-driven signaling in PC cells. We compared, via GSEA, the transcriptional footprint of dilazep with that of AR siRNA in androgen-dependent LNCaP and androgen-independent Abl cells. In both cell lines, genes downregulated by AR siRNA were strongly enriched among the genes suppressed by dilazep, while genes upregulated by AR siRNA were strongly enriched among the genes induced by dilazep in the same cell line. This provides evidence that dilazep indeed suppresses the AR axis, not only under androgen-dependent conditions but also in CRPC cells.

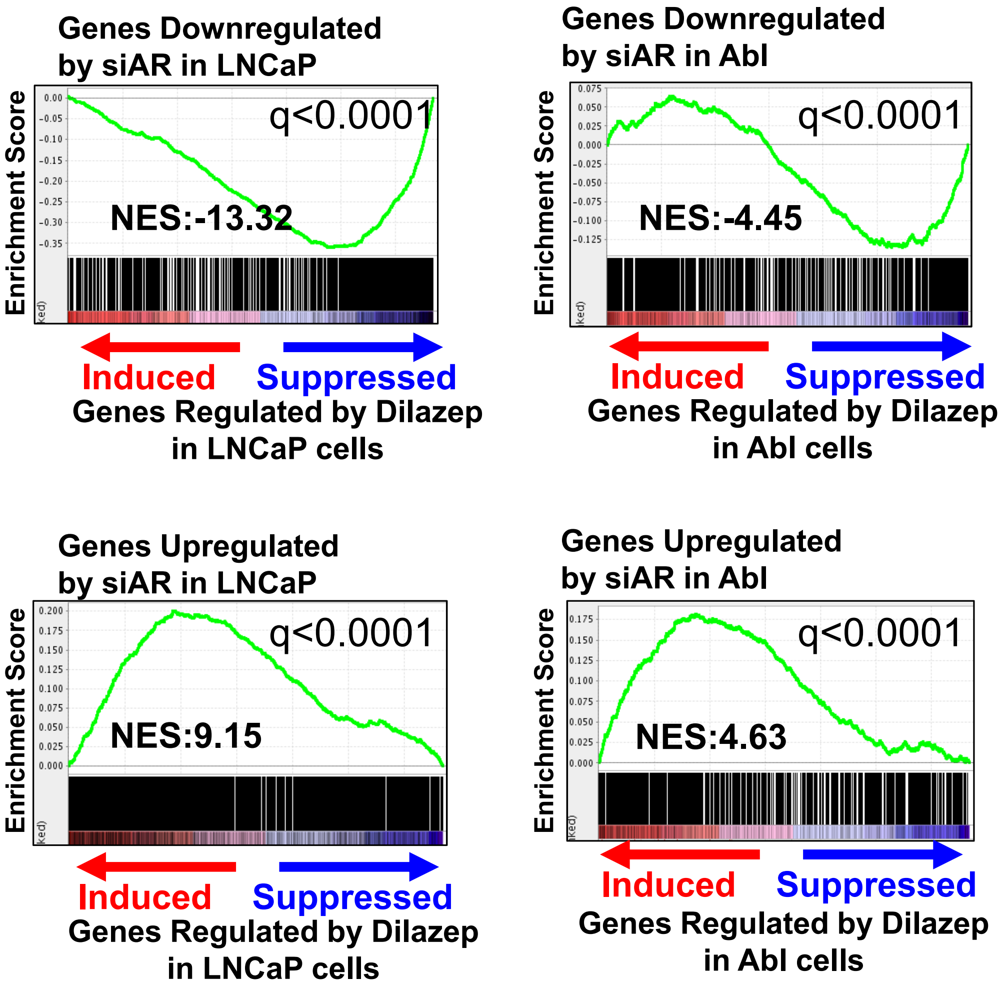

Supplement: Suppl. Fig. 6 Dilazep suppresses AR-driven signaling in PC cells. We compared, via GSEA, the transcriptional footprint of dilazep with that of AR siRNA in androgen-dependent LNCaP and androgen-independent Abl cells. In both cell lines, genes downregulated by AR siRNA were strongly enriched among the [file supplementary_figure_9.pdf]
