# Supplementary material for: Inhibition of GATA2 in prostate cancer by a clinically available small molecule
Source: Endocr Relat Cancer. 2021 Oct 12;29(1):15–31. doi: 10.1530/ERC-21-0085 (PMC8634153; doi:10.1530/ERC-21-0085)
Supplement: Suppl. Fig. 7 Dilazep treatment diminishes protein expression of AR, c-Myc and regulators of cell-cycle progression. Heatmap representation of selected proteins revealed to have been changed via RPPA analysis. LNCaP-MDVR cells were treated with 50 µM of dilazep (or H2O control) for 48 hrs and submit [file supplementary_figure_10.pdf]

Suppl. Fig. 7

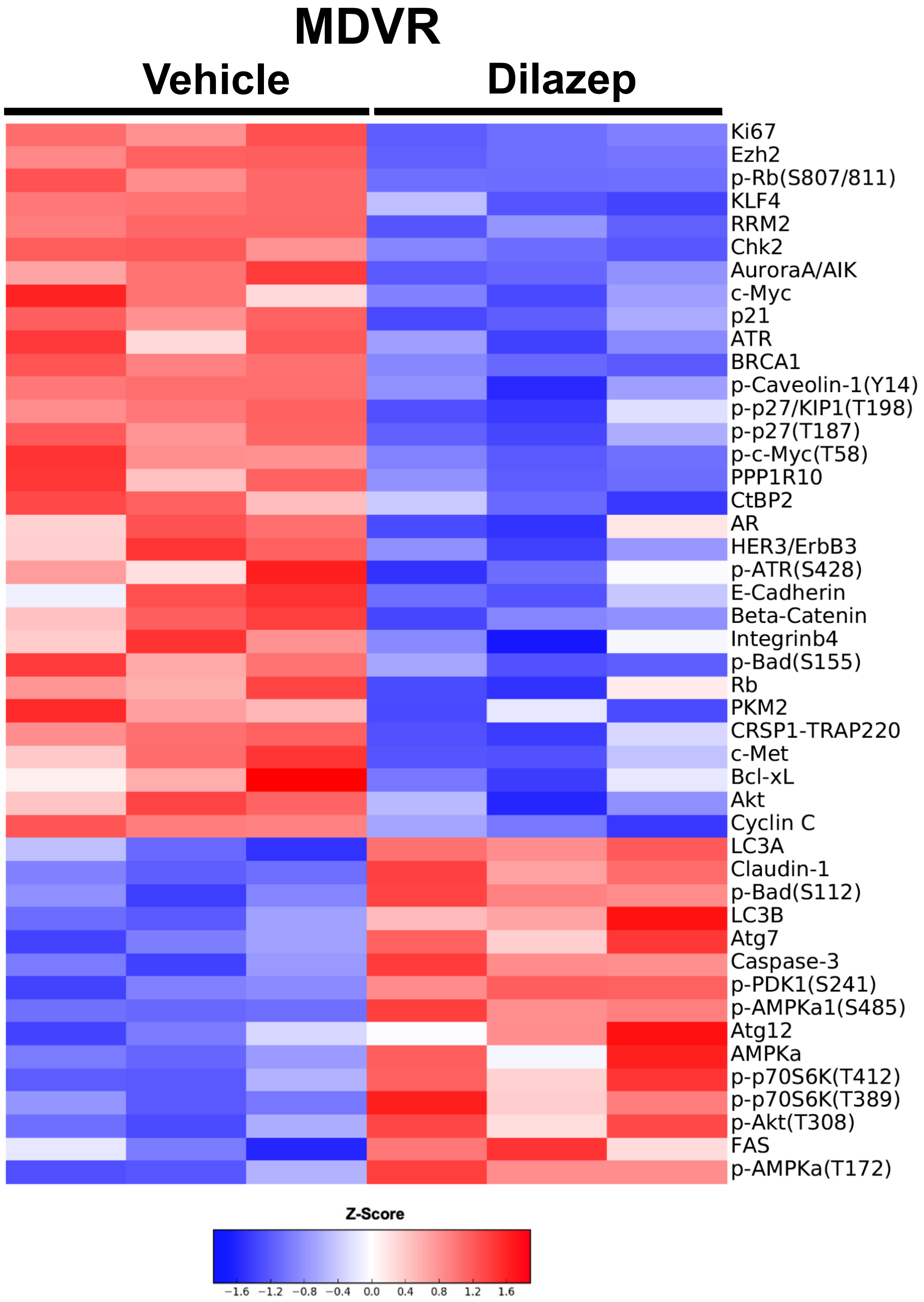

Dilazep treatment diminishes protein expression of AR, c-Myc and regulators of cell-cycle progression. Heatmap representation of selected proteins revealed to have been changed via RPPA analysis. LNCaP-MDVR cells were treated with 50 μM of dilazep (or H_2O control) for 48 hrs and submitted in triplicate for RPPA analysis. Heatmap reveals protein expression change, in log(2) scale, for dilazep treated cells compared to vehicle treated cells. The proteomic signature caused by dilazep treatment in MDVR cells was highly concordant with that seen in LNCaP and Abl cells (**Fig. 5**).
