# Supplementary figures and images for: Inhibition of GATA2 in prostate cancer by a clinically available small molecule
Source: Endocr Relat Cancer. 2021 Oct 12;29(1):15–31. doi: 10.1530/ERC-21-0085 (PMC8634153; doi:10.1530/ERC-21-0085)

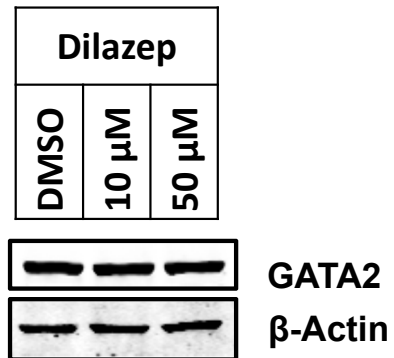

Dilazep treatment in LNCaP cells for 16 hrs does not suppress total GATA2 protein levels.

Supplement: Suppl. Fig. 8 Dilazep treatment in LNCaP cells for 16 hrs does not suppress total GATA2 protein levels. [file supplementary_figure_11.pdf]
