# Supplementary material for: Inhibition of GATA2 in prostate cancer by a clinically available small molecule
Source: Endocr Relat Cancer. 2021 Oct 12;29(1):15–31. doi: 10.1530/ERC-21-0085 (PMC8634153; doi:10.1530/ERC-21-0085)
Supplement: Supplementary Materials [file supplementary_material.pdf]

Supplementary Text

The PSA61-Luc plasmid (Cleutjens et. al. 1997 Molecular Endocrinology) carries a 6-kb fragment of the promoter/enhancer region of the PSA/KLK3 gene. This fragment harbors a 440-bp regulatory region (shown underlined) which contains three GATA2 binding motifs and one ARE. Two additional AREs and several GATA2 binding motifs are also present on this plasmid further downstream, prior to the luciferase gene.

The green letter A is the transcription start site of KLK3

Three AREs are shown highlighted in yellow.

GATA2 consensus binding sequences W/GATA/R, where W can be A or T and R can be A or G, are highlighted in turquoise.

5’ end of a sequence 6000bp upstream of KLK3 TSS:
CATCTCTGTGACTCATGTTATTGTACTTTAAAGTGGAAATAATAACAGTTCCTGTACAGTTTAGGGCTTCCCAAGATGACCCTCCATTGACATCAGTTGAGATT
CCAGGAGAGTCCAAAAGATCATCCTTAAGGTTCAGTCGTTTGCTAGGATGCTCATGGTTGTGGTTTATTACAGCAAAAGGATACAGATGAAAATCTGGCAAGGG
AGAGGTGAATGAGGCAAAGACAAGGAGAACTCAAAGTGCGAAGCTTCTAGTTTTCTTTTCCCGGTGACATCGTGGAAAGCACTAGCATCTCTAAGCAATGATCT
GTGACAATATTCACAGTGTATTGCCATCCAGGGAACTCAACTGAGCCTTGATGTCCAGAGATTTTTGTGTTTTTTTCTGAGACTGAGTCTCGCTCTGTGCCCAG
GCTGGAGTGCAGTGGTGCAACCTTGGCTCACTGCAAGCTCCGCCTCCTGGGTTCACGCCATTCTCCTGCCTCAGCCTCCTGAGTAGCTGGGACTACAGGCACCC
GCCACCACGCCTGGCTAATTTTTTTGTATTTTTAGTAGAGATGGGGTTTCACTGTGTTAGCCAGGATGGTCTCAGTCTCCTGACCTCGTGATCTGCCCACCTTG
GCCTCCCAAAGTGCTGGGATTACAGGCGTGAGCCACTGCGCCTGGCCGATATCCAGAGATTTTTTGGGGGGCTCCATCACACAGACATGTTGACTGTCTTCATG
GTTGACTTTTAGTATCCAGCCCCTCTAGAAATCTAGCTGATATAGTGTGGCTCAAAACCTTCAGCACAAATCACACCGTTAGA**ctatct**GGTGTGGCCCAAAC
CTTCAGGTGAACAAAGGCACTCTAAACTGGCAGGATATTCCAAAGCATTAGAGATGACCTCTTGCAAAGAAAAAGAAATGGAAAAGAAAAAGAAAGAAAGGAAA
AAAAAAAAAAAAAAGAGATGACCTCTCAGGCTCTGAGGGGAAACGCCTGAGGTCTTTGAGCAAGGTCAGTCCTCTGTTGCACAGTCTCCCTCACAGGGTCATTG
TGACGATCAAATGTGGTCACGTGTATGAGGCACCAGCACATGCCTGGCTCTGGGGAGTGCCGTGTAAGTGTATGCTTGCACTGCTGAATGGCTGGGATGTGTCA
GGGA**ttatct**TCAGCACTTACAGATGCTCATCTCATCCTCACAGCATCACTATGGGATGGGTATTACTGGCCTCATTTGATGGAGAAACTGGCTGTGGCTCAG
AAAGGGGGGACCACTAGACCAGGGACACTCTGGATGCTGGGGACTCCAGAGACCATGACCACTCACCAACTGCAGAGAAATTAATTGTGGCCTGATGTCCCTGT
CCTGGAGAGGGTGGAGGTGGACCTTCACTAACCTCCTACCTTGACCCTCTCTTTTAGGGCTCTTTCTGACCTCCACCATGATACTAGGACCCCATTGTATTCTG
TACCCTCTTGACTCTATGACCCCCACTGCCCACTGCATCCAGCTGGGTCCCCTC**ctatct**CTATTCCCAGCTGGCCAGTGCAGTCTCAGTGCCCACCTGTTTG
TCAGTAACTCTGAAGGGGCTGACATTTTACTGACTTGCAAACAAATAAGCTAACTTTCCAGAGTTTTGTGAATGCTGGCAGAGTCCATGAGACTCCTGAGTCAG
AGGCAAAGGCTTTTACTGCTCACAGCTTAGCAGACAGCATGAGGTTCATGTTCACATTAGTACACCTTGCCCCCCCCAAATCTTGTAGGGTGACCAGAGCAGTC
TAGGTGGATGCTGTGCACACGGGGTTTGTGCCACTGGTGAGAAACCTGAGATTAGGAATCCTCAATCTTATACTGGGACAACTTGCAAACCTGCTCAGCCTTTG
TCTCTGATGAAGATA**ttatct**TCATGATCTTGGATTGAAAACAGACCTACTCTGGAGGAACATATTGTATCGATTGTCCTTGACAGTAAACAAATCTGTTGTA
AGAGACA**ttatct**TTA**ttatct**AGGACAGTAAGCAAGCCTGGATCTGAGAGAGATATCATCTTGCAAGGATGCCTGCTTTACAAACATCCTTGAAACAACAAT
CCAGAAAAAAAAAAGGTGTTGCTGTCTTTGCTCAGAAGACACACAGATACGTGACAGAACCATGGAGAATTGCCTCCCAACACTGTTCAGCCAGAGCCTTCCACC
CTTGTCTGCAGGACAGTCTCAACGTTCCACCATTAAATACTTCTTCTGTCACATCCTGCTTATTTATGCCTAACCAAGGTTCTAGGTCCCGATCGACTGTGTCT
GGCAGCACTCCACTGCCAAACCCAGAATAAGGCAGCGCTCAGGATCCCGAAGGGGCATGGCTGGGGATCAGAACTTCTGGGTTTGAGTGAGGAGTGGGTCCACC
CTCTTGAATTTCAAAGGAGGAAGAGGCTGGATGTGAAGGAACTGGGGGAGGGAAAGTGTCAGTTCCGAACTCTTAGGTCAATGAGGGAGGAGACTGGTAAGGTC
CCAGCTCCCGAGGTACTGATGTGGGAATGGCCTAAGAATCTCATATCCTCAGGAAGAAGGTGCTGGAATCCTGAGGGGTAGAGTTCTGGGTATATTTGTGGCTT
AAGGCTCTTTGGCCCCTGAAGGGCAGAGGCTGGAACCATTAGGTCCAGGGTTTGGGG**tgatag**TAATGGGATCTCTTGATTCCTCAAGAGTCTGAGGATCGAG
GGTTGCCCATTCTTCCATCTTGCCACCTAATCCTTACTCCACTTGAGGGTATCACCAGCCCTTCTAGCTCCATGAAGGTGCCCCTGGGCAAGCACAATCTGAGC
ATGAAAGATGCCCCAGAGGCCTTGGGTGTCATCCACTCATCATCCAGCATCCACACTCTGAGGGTGTGGCCAGCACCATGACGTCATGTTGCTGTGACTATCCC
TGCAGCGTGCCTCTCCAGCCACCTGCCAACCGTAGAGCTGCCGACATCCTCCTCTGGTGGGAGTGGCCTGCATGGTGCCAGGCTGAGGCCTAGTGTCAGACAGG
GAGCCTGGAATCATAGGGATCCAGGACTCAAAAGTGCTAGAGAATGGCCATATGTCACCATCCATGAAATCTCAAGGGCTTCTGGGTGGAGGGCACAGGGACCT
GAACTTATGGGTTTTCCCCAAGTCTATTGCTCTCCCAAGTGAGTCTCCCAGATACGAGGCACTGTGCCAGCATCAGCC**ttatct**CCACCACATCTTGTAAAAG
GGACTACCCAGGGCCCTGATGAACACCATGGTGTGTACAGGAGTAGGGGGTGGAGGCACGGACTCCTGTGAGGTCACAGCCAAGGGAGCATCATCATGGGTGGG
GAGGAGGCAATGGACAGGCTTGAGAACGGGGATGTGGTTGTATTTGGTTTTCTTTGGTT**agataa**AGTGCTGGGTATAGGATTGAGAGTGGAGTATGAAGACC

AGTTAGGATGGAGGATCAGATTGGAGTTGGGTTAGAGATGGGGTAAAATTGTGCTTCGGATGAGTTTGGGATTGACACTGTGGAGGTGGTTTGGGATGGCATGG
CTTTGGGATGGAAATAGATTTGTTTTGATGTTGGCTCAGACATCCTTGGGGATTGAACTGGGGATGAAGCTGGGTTTGATTTTGGAGGTAGAAGACGTGGAAGT
AGCTGTCAGATTTGACAGTGGCCATGAGTTTTGTTTGATGGGGAATCAAACAATGGGGGAAGACATAAGGGTTGGCTTGTTAGGTTAAGTTGCGTTGGGTTGAT
GGGGTCGGGGCTGTGTATAATGCAGTTGGATTGGTTTGTATTAAATTGGGTTGGGTCAGGTTTTGGTTGAGGATGAGTTGAGGATATGCTTGGGGACACCGGAT
CCATGAGGTTCTCACTGGAGTGGAGACAAACTTCCTTTCCAGGATGAATCCGGGGAAGCCTTAATTCACGTGTAGGGGAGGTCAGGCCACTGGCTAAGTATATC
CTTCCACTCCAGCTCTAAGATGGTCTTAAATTGTGA**ttatct**ATATCCACCTCTGTCTCCCTCACTGTGCTTGGAGTTTACCTGATCACTCAACTAGAAACAG
GGGAAGATT**ttatca**AATTCTTTTTTTTTTTTTTTTTTTTTTTGAGACAGAGTCTCACTCTGTTGCCCAGGCTGGAGTGCAGTGGCGCAGTCTCGGCTCACTG
CAACCTCTGCCTCCCAGGTTCAAGTGATTCTCCTGCCTCAGCCTCCTGAGTTGCTGGGATTACAGGCATGCAGCACCATGCCCAGCTAATTTTTGTATTTTTAG
TAGAGATGGGGTTTCACCAATGTTTGCCAGGCTGGCCTCGAACTCCTGACCTGGTGATCCACCTGCCTCAGCCTCCCAAAGTGCTGGGATTACAGGCGTCAGCC
ACCGCGCCCAGCCACTTTTGTCAAATTCTTGAGACACAGCTCGGGCTGGATCAAGTGAGCTACTCTGGTTTTATTGAACAGCTGAAATAACCAACTTTTTGGAA
ATTGATGAAATCTTACGGAGTTAACAGTGGAGGTACCAGGGCTCTTAAGAGTTCCCGATTCTCTTCTGAGACTACAAATTGTGATTTTGCATGCCACCTTAATC
TTTTTTTTTTTTTTTTTAAATCGAGGTTTCAGTCTCATTCTATTTCCCAGGCTGGAGTTCAATGGCGTGATCACAGCTCACTGTAGCCTTGAACTCCTGGCCTT
AAGAGATTCTCCTGCTTCGGTCTCCCAATAGCTAAGACTACAGTAGTCCACCACCATATCC**agataa**TTTTTAAATTTTTTGGGGGGCCGGGCACAGTGGCTC
ACGCCTGTAATCCCAACACCATGGGAGGCTGAGATGGGTGGATCACGAGGTCAGGAGTTTGAGACCAGCCTGACCAACATGGTGAAACTCTGTCTCTACTAAAA
AAAAAAAAAATAGAAAAATTAGCCGGGCGTGGTGGCACACGGCACCTGTAATCCCAGCTACTGAGGAGGCTGAGGCAGGAGAATCACTTGAACCCAGAAGGCAG
AGGTTGCAATGAGCCGAGATTGCGCCACTGCACTCCAGCCTGGGTGACAGAGTGAGACTCTGTCTCAAAAAAAAAAAAATTTTTTTTTTTTTTTGTAGAGATGG
ATCTTGCTTTGTTTCTCTGGTTGGCCTTGAACTCCTGGCTTCAAGTGATCCTCCTACCTTGGCCTCGGAAAGTGTTGGGATTACAGGCGTGAGCCACCATGACT
GACCTGTCGTTTAATCTTGAGGTACATAAACCTGGCTCCTAAAGGCTAAATATTTTGTTGGAGAAGGGGCATTGGATTTTGCATGAGGATGATTCTGACCTGGG
AGGGCAGGTCAGCAGGCATCTCTGTTGCAC**agatag**AGTGCACAGGTCTGGAGAACAAGGAGTGGGGGGTTATTGGAATTCCACATTGTTTGCTGCACGTTGG
ATTTTGAAATGCTAGGGAACTTTGGGAGACTCATATTTCTGGGCTAGAGGATCTGTGGACCACAAGATCTTTTTATGATGACAGTAGCAATGTATCTGTGGAGC
TGGATTCTGGGTTGGGAGTGCAAGGAAAAGAATGTACTAAATGCCAAGACATCTATTTCAGGAGCATGAGGAATAAAAGTTCTAGTTTCTGGTCTCAGAGTGGT
GCAGGGATCAGGGAGTCTCACAATCTCCTGAGTGCTGGTGTCTTAGGGCACACTGGGTCTTGGAGTGCAAAGGATCTAGGCACGTGAGGCTTTGTATGAAGAAT
CGGGGATCGTACCCACCCCCTGTTTCTGTTTCATCCTGGGCGTGTCTCCTCTGCCTTTGTCCCCTAGATGAAGTCTCCATGAGCTACAGGGCCTGGTGCATCCA
GGGTGATCTAGTAATTGCAGAACAGCAAGTGCTAGCTCTCCCTCCCCTTCCACAGCTCTGGGTGTGGGAGGGGGTTGTCCAGCCTCCAGCAGCATGGGGAGGGC
CTTGGTCAGCCTCTGGGTGCCAGCAGGGCAGGGGCGGAGTCCTGGGGAATGAAGGTTTTATAGGGCTCCTGGGGGAGGCTCCCC**A**
